# HOXD9/miR-451a/PSMB8 axis is implicated in the regulation of cell proliferation and metastasis via PI3K/AKT signaling pathway in human anaplastic thyroid carcinoma

**DOI:** 10.1186/s12967-023-04538-0

**Published:** 2023-11-16

**Authors:** Yong Zhong, Fan Yu, Ling Yang, Yu Wang, Lin Liu, Chengyou Jia, Haidong Cai, Jianshe Yang, Shiyang Sheng, Zhongwei Lv, Li Weng, Bo Wu, Xiaoping Zhang

**Affiliations:** 1 Department of Nuclear Medicine, Shanghai Tenth People’s Hospital, Tongji University, Shanghai, 200072 China; 2 Department of General Surgery, Shanghai Sixth People’s Hospital Affiliated to Shanghai Jiao Tong University School of Medicine, 600 Yishan Road, Shanghai, 200233 China; 3 Department of Head and Neck Surgery, Fudan University Shanghai Cancer Center, Shanghai, China; 4 Department of Intervention, Tongren Hospital, Shanghai Jiao Tong University School of Medicine, Shanghai, 200336 China; 5 Department of Nuclear Medicine, Shanghai Tenth People’s Hospital, Tongji University and Shanghai Center of Thyroid Diseases, No. 301 Middle Yanchang Road, Shanghai, 200072 China; 6 Center of Thyroid, Department of General Surgery, Shanghai Jiao Tong University Affiliated Sixth People’s Hospital, Shanghai, 200233 China

**Keywords:** Anaplastic thyroid carcinoma, HOXD9, miR-451a, PSMB8, Metastasis, Proliferation, EMT, PI3K/AKT pathway

## Abstract

Anaplastic thyroid carcinoma (ATC) is a deadly disease with a poor prognosis. Thus, there is a pressing need to determine the mechanism of ATC progression. The homeobox D9 (HOXD9) transcription factor has been associated with numerous malignancies but its role in ATC is unclear. In the present study, the carcinogenic potential of HOXD9 in ATC was investigated. We assessed the differential expression of HOXD9 on cell proliferation, migration, invasion, apoptosis, and epithelial–mesenchymal transition (EMT) in ATC and explored the interactions between HOXD9, microRNA-451a (miR-451a), and proteasome 20S subunit beta 8 (PSMB8). In addition, subcutaneous tumorigenesis and lung metastasis in mouse models were established to investigate the role of HOXD9 in ATC progression and metastasis in vivo. HOXD9 expression was enhanced in ATC tissues and cells. Knockdown of HOXD9 inhibited cell proliferation, migration, invasion, and EMT but increased apoptosis in ATC cells. The UCSC Genome Browser and JASPAR database identified HOXD9 as an upstream regulator of miR-451a. The direct binding of miR-451a to the untranslated region (3′-UTR) of PSMB8 was established using a luciferase experiment. Blocking or activation of PI3K by LY294002 or 740Y-P could attenuate the effect of HOXD9 interference or overexpression on ATC progression. The PI3K/AKT signaling pathway was involved in HOXD9-stimulated ATC cell proliferation and EMT. Consistent with in vitro findings, the downregulation of HOXD9 in ATC cells impeded tumor growth and lung metastasis in vivo. Our research suggests that through PI3K/AKT signaling, the HOXD9/miR-451a/PSMB8 axis may have significance in the control of cell proliferation and metastasis in ATC. Thus, HOXD9 could serve as a potential target for the diagnosis of ATC.

**Graphical Abstract:**

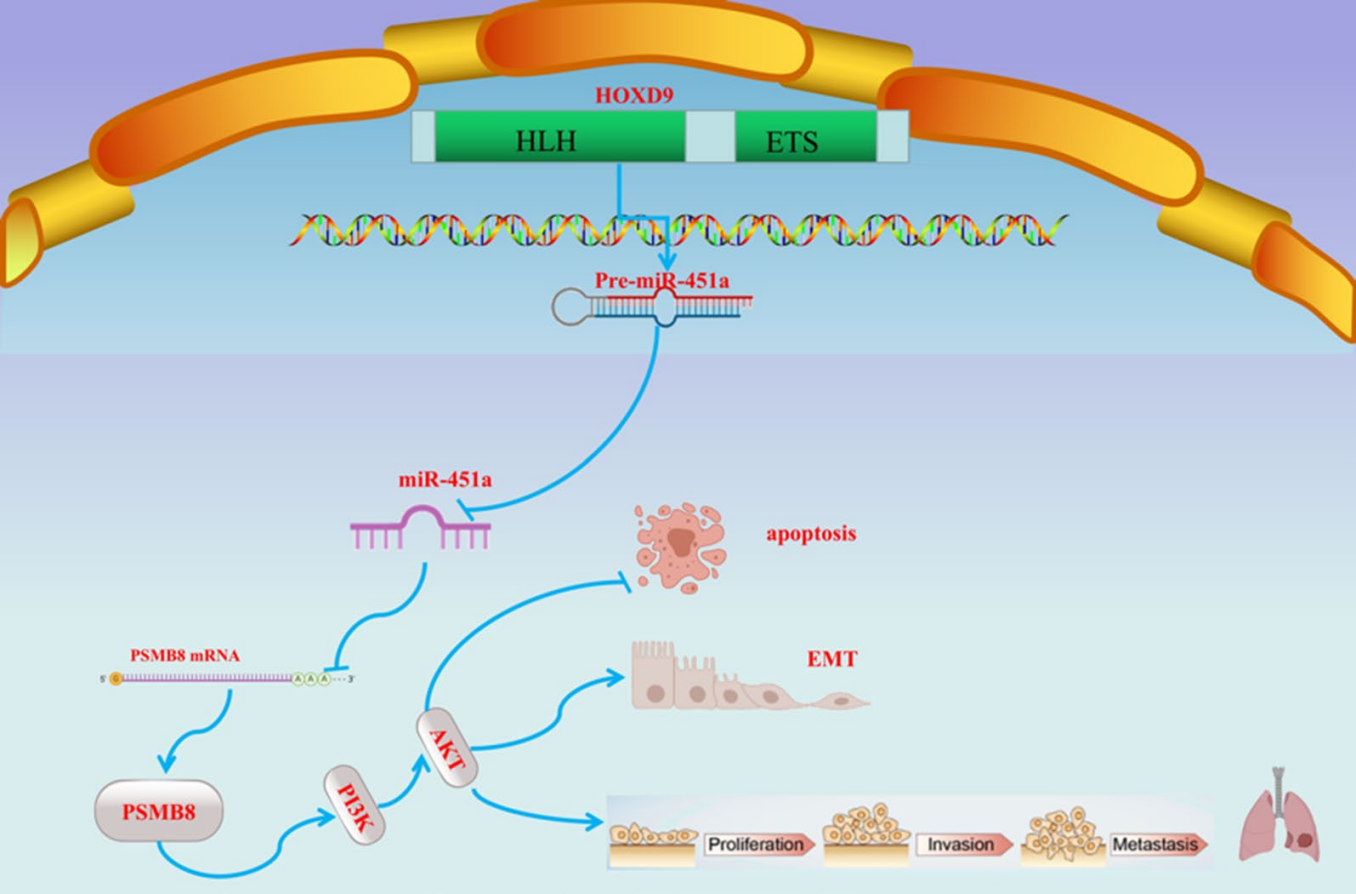

**Supplementary Information:**

The online version contains supplementary material available at 10.1186/s12967-023-04538-0.

## Introduction

Anaplastic thyroid carcinoma (ATC) is a rare malignancy accounting for only 1–2% of all thyroid cancers but its occurrence signifies a dismal prognosis and results in the majority of thyroid carcinoma deaths [1, 2]. ATC is a highly aggressive cancer characterized by a high Ki-67 proliferative rate and with median survival times of only 4 months [1]. In contrast to differentiated thyroid cancers that have a relatively good prognosis, ATC is associated with a rapidly growing invasive tumor with distant metastases that does not respond well to surgical intervention [3]. A better understanding of the molecular progression of the disease is the key to finding an effective treatment for ATC.

The homeobox (HOX) genes are a family of transcription factors associated with several cellular processes including proliferation, apoptosis, and migration [4, 5]. The homeoproteins that are encoded by HOX genes contain a conserved domain that regulates the expression of several target genes [6]. The aberrant regulation of homeoprotein target genes can give rise to cancer and several are associated with metastases [7, 8]. Particularly, HOXD9 is associated with several cancers, including gliomas, esophageal cancer, gastric cancer, and hepatocellular carcinoma [9–12]. In hepatocellular carcinoma (HCC), HOXD9 was found to promote epithelial–mesenchymal transition (EMT) by interacting with the promoter of *ZEB1* [11]. Whereas in gastric cancer, HOXD9 could influence the proliferation, migration, and invasiveness of cells by interacting with the promoter of *RUFY3* [12]. However, whether HOXD9 regulates the invasive and migratory characteristics of ATC is unclear.

Several studies have implicated EMT in the development of ATC and metastases [13, 14]. EMT is identified in ATC by several characteristic markers, which include increased expression of N-cadherin and a decrease in the expression of E-cadherin and Vimentin [15]. During the EMT process, the number of stem cells is elevated and contributes to the proliferative and metastatic characteristics of ATC [16]. In ATC, EMT is regulated by several microRNAs (miRNAs), such as the miR-200 family [17], which are associated with various key genes and pathways. For instance, miR-205 downregulates HOXD9 to suppress EMT in human glioma [18]. In this study, we found that HOXD9 was an upstream regulator of miR-451a. The development of several cancers is associated with the differential expression of miR-451a, including thyroid cancers [19, 20]. MiR-451a is also involved in the regulation of EMT [21, 22]. For instance, the levels of miR-451a are downregulated in cutaneous squamous cell carcinoma, which results in increased EMT through the upregulation of the PI3K/AKT signaling pathway [21]. However, it is unclear whether miR-451a has a similar role in ATC.

Proteasome subunit beta type-8 (PSMB8), also known as the large multifunctional protease 7, encodes β5i which often functions as a subunit of the immunoproteasome [23]. A previous study found that PSMB8 overexpression correlated with the advancement of gastric cancer, particularly in characteristics linked to tumor invasion and lymph node metastases [24]. However, there is a paucity of information associated with the specific molecular mechanisms of PSMB8 in many disorders, and the molecular mechanisms of PSMB8 in ATC are not known at all.

In this study, we investigated the carcinogenic potential of HOXD9 in ATC and its possible interactions with miR-451a and PSMB8. In particular, we determined whether the HOXD9/miR-451a/PSMB8 axis was implicated in the regulation of ATC cell proliferation and metastasis in cell cultures and a murine model. In addition, PI3K inhibitors and agonists were used to further investigate whether the PI3K/AKT signaling pathway was involved in HOXD9/miR-451a/PSMB8 axis-mediated ATC cell proliferation and metastasis. Our findings suggest that HOXD9 has pro-tumor activity in ATC and acts as a cancer promoter. The HOXD9/miR-451a/PSMB8 axis promotes ATC cell growth, migration, invasion, and EMT by activating the PI3K/AKT signaling pathway.

## Materials and methods

### Patient tissues

ATC tissues and adjacent normal tissues were obtained from patients after resection of thyroid carcinomas or thyroid nodules between 2010 and 2019 at the Fudan University Shanghai Cancer Center and Shanghai Jiao Tong University Affiliated Sixth People’s Hospital (Shanghai, China). Patients included 28 males and 12 females from 30 to 86 years old. Pre-operative chemotherapy was not performed on any of the patients. The Research Ethics Committee of Fudan University Shanghai Cancer Center and Shanghai Jiao Tong University Affiliated Sixth People’s Hospital approved the research. The clinical ethics number is 2022-KY-12(K), and the approval date was 2022-01-27. All cancerous tissue specimens, adjacent tissues with no cancer cells, and inflammatory cell infiltration were diagnosed by pathology. The diagnosis in tissue sections and tissue variants was confirmed by three pathologists. Tissues were resected, stored in liquid nitrogen, and then stored at −  80 ℃ for RNA extraction.

### Cell culture

The human ATC cell lines, 8505c, FRO, and HTH7, and the thyroid follicular cell line Nthy-ori 3–1 were purchased from the American Type Culture Collection (ATCC, Manassas, VA, USA) and the Shanghai Institute of Cell Biology (Shanghai, China). The Nthy-ori 3–1 cells were cultured in RPMI-1640 medium (Gibco, Carlsbad, CA, USA) and the ATC cell lines were cultured in Dulbecco’s modified Eagle’s medium (DMEM, Gibco). All cultures included the addition of 10% fetal bovine serum (FBS, Hyclone, Logan, UT, USA) and 100 U/mL penicillin/streptomycin (Gibco). Cells were grown at 37 ℃ in an atmosphere of 5% CO_2_. For activation and suppression of the PI3K-Akt signaling pathway, cells were exposed to 740Y-P (activator of PI3K signaling, 30 μM, #HY-P0175, MCE, Monmouth Junction, NJ, USA) or LY294002 (inhibitor of PI3K signaling, 25 μM, #HY-10108, MCE).

### In vivo* tumor growth assay*

All animal studies were approved by the Ethics Committee of Shanghai Tenth People’s Hospital of China (Shanghai, China). The animal experiments were designed using the Principles of Laboratory Animal Care (National Society for Medical Research) and the National Institutes of Health guidelines, and the animal research ethics number was SHDSYY-2021-1811117. Untreated and si-NC or si-HOXD9 transfected 8505c cells (1 × 10^7^ in 100 μL PBS) were injected subcutaneously in the right hind flank of 6-week-old BABL/c-nu female mice (Shanghai Institute of Laboratory Animals, Chinese Academy of Sciences). Mice were randomly allocated into one of three treatment groups (n = 6/group) and housed in specific pathogen-free conditions with free access to food and water. To evaluate tumor growth, the mice carrying HOXD9 siRNA or scramble siRNA cells were intraperitoneally administered with 150 mg/kg body weight D-luciferin (Caliper Life Sciences, Waltham, MA, USA), and the cells emitted a visual light signal that was monitored using an in vivo imaging system (IVIS, Xenogen Corp., Alameda, CA, USA). The formula for measuring the tumors was: volume = (width^2^ × length)/2 and tumor volume was calculated every 3 days. The mice were euthanized 24 days after the injection and the tumors were excised, weighed, and photographed. The average size of the tumors in each experiment group at set times was used to plot growth curves. Samples from the tumors were randomly selected for western blotting, TUNEL staining, and immunohistochemistry.

### In vivo* metastasis model*

Mice were divided into three groups (n = 6): untreated mice, scrambled siRNA-treated mice, and HOXD9 siRNA-treated mice. To investigate the effect of HOXD9 on metastasis in vivo, female 6-week-old SCID mice (Shanghai Institute of Laboratory Animals, Chinese Academy of Sciences, Shanghai, China) were injected with 5 × 10^5^ 8505c-luc cells infected with HOXD9-siRNA or siRNA scramble through the lateral tail vein. The mice were monitored for general health status. D-luciferin (150 mg/kg body weight) was used to monitor the presence of distant lung metastases using an IVIS. On days 2, 8, 14, and 28, images of tumor metastasis in the lung were obtained by using the IVIS. At 4 weeks post-infection, recipient mice were euthanized and the lungs were dissected from the mice and weighed. After removal, the lungs were photographed and tumor colonies were counted. Lungs with visible tumor colonies were fixed and embedded in paraffin, and three non-sequential sections per animal were obtained. These samples were also analyzed with western blotting, H&E, and immunohistochemistry in addition to histological examination.

### Plasmid construction and siRNA interference assay

Mammalian expression plasmids designed to specifically express the full-length open reading frame of the human HOXD9 and PSMB8 genes were purchased from Genescript (Nanjing, China). An empty plasmid served as a negative control (control plasmid). Three pairs of small interfering RNAs (siRNAs) were designed to knock down the expression of HOXD9, E-cadherin, and PSMB8. All siRNAs were synthesized by Ribobio (Guangzhou, China). The targeting sequences are listed in Additional file 1: Table S1. The 8505c cells were grown to 60–75% confluence in six-well plates. The overexpression plasmids and siRNAs were transfected into 8505c cells for 24 to 48 h using Lipofectamine 3000 (Invitrogen, Carlsbad, CA, USA), according to the manufacturer’s instructions.

### Transfection with miR-451a mimics and inhibitors

The overexpression and knockdown of miRNA were achieved by transfecting 8505c cells with miRNA mimics or inhibitors, respectively. Synthetic miRNA mimics, inhibitors, and scrambled negative control RNAs (inhibitor NC and mimic NC) were purchased from GenePharma, Shanghai, China. 8505c cells were seeded in six-well plates 24 h before miR-451a mimic or inhibitor transfection with 50–60% confluence and then were transfected with Lipofectamine 2000 (Invitrogen) following the manufacturer's protocol. At 6 h after transfection, the 8505c cell medium was changed to DMEM supplemented with 2% FBS. The cells were harvested 48 h after transfection for total RNA or protein isolation.

### Real-time quantitative PCR (qRT-PCR)

The total RNA was isolated from cells and cancer tissues using Trizol reagent (Invitrogen). An SYBR Premium Ex Taq II Kit (Takara, Dalian, China) and the ABI PRISM 7500 Sequence Detection System (Applied Biosystems, Foster City, CA, USA) were used to perform qRT-PCR. The primers are listed in Table 1. The 2^−ΔΔCT^ method was used to measure levels of expression relative to glyceraldehyde 3-phosphate dehydrogenase (GAPDH) or small nuclear RNA U6.

**Table 1:** Primers used for qRT-PCR in this study

Gene	Sense	Antisense
HOXD9	CAGTGGTTTGACGGGGTGAT	GTCGTGGGCCTGTTGCTTAT
PSMB8	GCTGCGCCTTTAGATGACAC	TCCACTGCTGCAATCACTCC
miR-451a	CGCGAAACCGTTACCATTAC	AGTGCAGGGTCCGAGGTATT
U6	GATTATCGGGACCATTCCACTG	GATCTGGTTCCCAATGACTGTG
PCNA	TGTTGGAGGCACTCAAGGAC	GAGTCCATGCTCTGCAGGTT
Ki-67	TGCCCGACCCTACAAAATG	GAGCCTGTATCACTCATCTGC
CCNA2	CTGCATTTGGCTGTGAACTAC	ACAAACTCTGCTACTTCTGGG
CCNB1	GGCTTTCTCTGATGTAATTCTTGC	GTATTTTGGTCTGACTGCTTGC
GAPDH	CGAGCCACATCGCTCAGACA	GTGGTGAAGACGCCAGTGGA

### Western blotting

Cells and tissue were lysed with radioimmunoprecipitation assay buffer containing protease inhibitors. Proteins in the lysate were separated using SDS-PAGE and transferred to nitrocellulose membranes for western blotting. Membranes were blocked with 5% non-fat milk and then incubated with primary antibodies overnight at 4 ℃. The following antibodies were used: Anti-HOXD9 (Santa Cruz, sc-137134, 1:1,000), anti-PSMB8 (Abcam, Cambridge, UK, ab232984, 1:1,500), anti-GAPDH (Abcam, ab8245, 1:2,000), anti-N-cadherin (Abcam, ab76011, 1:1,500), anti-E-cadherin (Abcam, ab40772, 1:1,000), anti-MMP7 (Abcam, ab216631, 1:1,000), anti-Vimentin (Abcam, ab92547, 1:1,000), anti-cleaved-caspase-3 (Cell Signaling, Danvers, MA, USA, #9661, 1:1,000), anti-Bcl-2 (Abcam, ab32124, 1:1,000), anti-Bax (Abcam, ab32503, 1: 1,500), and anti-Bid (Abcam, ab10640, 1: 1,000). After incubation, the membranes were washed and then treated for 1 h at 37 ℃ with goat antirabbit IgG conjugated with horseradish peroxidase (HRP). Chemiluminescence HRP substrate was used to visualize protein bands on the membrane. The gray values of protein bands were measured by Image J software (v1.8.0, NIH Image, Bethesda, MD, USA), with GAPDH as an internal control.

### Immunohistochemistry (IHC)

IHC with antigen retrieval was performed on deparaffinized sections. Sections were first blocked with 5% normal goat serum for 1 h at room temperature. Then they were incubated with anti-HOXD9 (Santa Cruz, sc-137134, 1: 500), anti-PSMB8 (Abcam, ab232984, 1:500), anti-CD31 (Abcam, ab182981, 1:500), anti-Ki-67 (Abcam, ab15580, 1: 500), anti-E-cadherin (Abcam, ab40772, 1: 1,000), and anti-N-cadherin (Abcam, ab76011, 1:500) primary antibodies for 1 h at room temperature. Positive signals in the tissue samples were detected using 3, 3-diaminobenzidine tetrahydrochloride substrate.

### Luciferase reporter assay

Luciferase reporter plasmids, pcDNA-PSMB8 or pcDNA-PSMB8-mut, were designed and constructed by Generay (Shanghai, China). For the luciferase reporter assay, 8505c cells were seeded and co-transfected with reporter plasmids and miR-451a mimic control or mimic using Lipofectamine 3000 transfection reagent (Thermo Fisher Scientific, Waltham, MA, USA). Luciferase activity was calculated by using the Dual-Luciferase Reporter Assay System (Promega, Madison, WI, USA) according to the manufacturer’s instructions. Firefly luciferase activity was detected and normalized against Renilla luciferase activity.

### TUNEL staining and apoptosis assay by flow cytometry

To determine the level of apoptosis, deparaffinized tissue sections were stained with terminal deoxynucleotidyl transferase dUTP nick end labeling (TUNEL) kits (BD Bioscience, Franklin Lakes, NJ, USA). The tissue sections were incubated with fluorescein isothiocyanate (FITC)-labeled TdT nucleotide mix at 37 ℃ for 60 min. The sections were counterstained with 10 mg/mL DAPI and positive cells were expressed as a percentage of the total cells stained. We also performed an apoptosis assay using flow cytometry as described previously [25]. Briefly, the cells (3 × 10^5^ cells/well) were seeded in a six-well plate and incubated with a combination of 5 μL Annexin V-FITC and 10 μL propidium iodide (PI) in the dark for 15 min in the dark at 25 ℃. BD FACSCalibur flow cytometry and CellQuest software (BD Bioscience) were used to measure Annexin V-FITC and PI fluorescence.

### Chromatin immunoprecipitation (ChIP) assay

We used an EZ-ChIP Chromatin Immunoprecipitation Kit (Millipore) to determine the interaction between the promoter region of miR-451a and HOXD9 in 8505c cells. Briefly, cells were fixed with 37% formaldehyde, lysed, and sonicated to shear DNA. The lysates were incubated with antibodies against HOXD9 (Abcam) or IgG overnight at 4 ℃. Protein A/G agarose beads were added, and the lysates were incubated at 4 ℃ for a further 1 h. They were then centrifuged at 5,000 *g* for 1 min at 4 ℃ and eluted with 20% SDS and 1 M NaHCO_3_ for 15 min at room temperature. Protein–DNA complexes were separated with 5 M NaCl overnight at 65 ℃, followed by 1 M Tris–HCl, 0.5 M EDTA, and proteinase K at 45 ℃ for 2 h. DNA was extracted using a spin column and the ChIP product was verified by PCR.

### CCK-8 assay

At 24 h before the experiment, cells in the logarithmic growth stage were transferred to 96-well plates at a density of 5 × 10^3^ cells per well, and the plates were placed in an incubator at 37 ℃ for a period of 72 h. After that, 10 µL of CCK-8 solution was added to each well, and the plates were placed in an incubator at 37 ℃ for a period of 2 h. The absorbance was measured at 450 nm using a microplate reader (Epoch Microplate Spectrophotometer, BioTek, Winooski, VT, USA). The experiments were performed in triplicates.

### Transwell migration and Matrigel invasion assays

The migration and invasion of cells were measured by using a Transwell assay (Corning Incorporated, Corning, NY, USA) in 24-well plates according to the manufacturer’s instructions. Briefly, a Matrigel matrix (1:5 dilution, 50 μL/well, BD Biosciences) was coated onto the Transwell membrane (8 μm pore size, 6.5 mm diameter) and used for the cell invasion assay. Briefly, ATC cells were plated in 24-well inserts (BD Bioscience) that were either coated in Matrigel for invasion assays or uncoated for migration assays. Cells (2 × 10^4^) in the upper chamber were grown in 100 μL serum-free medium whereas the lower chamber contained 600 μL 10% FBS. The cells were incubated for 24 h. Cells that migrated or invaded were counted in five random fields of each filter under a microscope at 200 × magnification.

### Colony formation assays

Colony formation by 8505c cells was examined by anchorage-independent soft agar assay. Briefly, 1.5 mL FBS-supplemented medium containing 0.5% agarose was poured into 35 mm cell culture dishes and allowed to solidify. Cells (5 × 10^3^ cells/well) were mixed with 1.5 mL FBS-supplemented medium containing 0.35% agarose and added to the top of the base agar. The cells were then cultured for 14 day at 37 ℃ under 5% CO_2_. The dishes were stained with 0.005% crystal violet (Sigma-Aldrich, St. Louis, MO, USA), and the colonies were examined with a microscope and digital camera.

### Statistical analysis

Statistical analysis of the data was performed using SPSS software (v16.0, Chicago, IL, USA) and GraphPad Prism (v 9.0, La Jolla, CA, USA). Data from three independent experiments were exhibited as mean value ± standard deviation (SD). An unpaired t-test was used to determine the significance between two groups. Two-way or one-way ANOVA followed by Tukey's post hoc test was applied for comparisons among multiple groups. Further statistical analysis was performed using the chi-square test for categorical variables and the Mann–Whitney U-test for continuous nonparametric variables. The relationship between HOXD9, miR-451a, or PSMB8 expression and the clinicopathological characteristics of ATC patients was evaluated using the chi-square test. Pearson correlation analysis was performed to analyze the correlations among the cancer-associated genes in clinical tissues. Cumulative survival probabilities were estimated by Kaplan–Meier methodology and differences in the survival rates between groups were calculated by log-rank testing. *P* values < 0.05 were considered statistically significant.

## Results

### HOXD9 is upregulated in ATC and correlates with poor prognosis

**Fig. 1:**
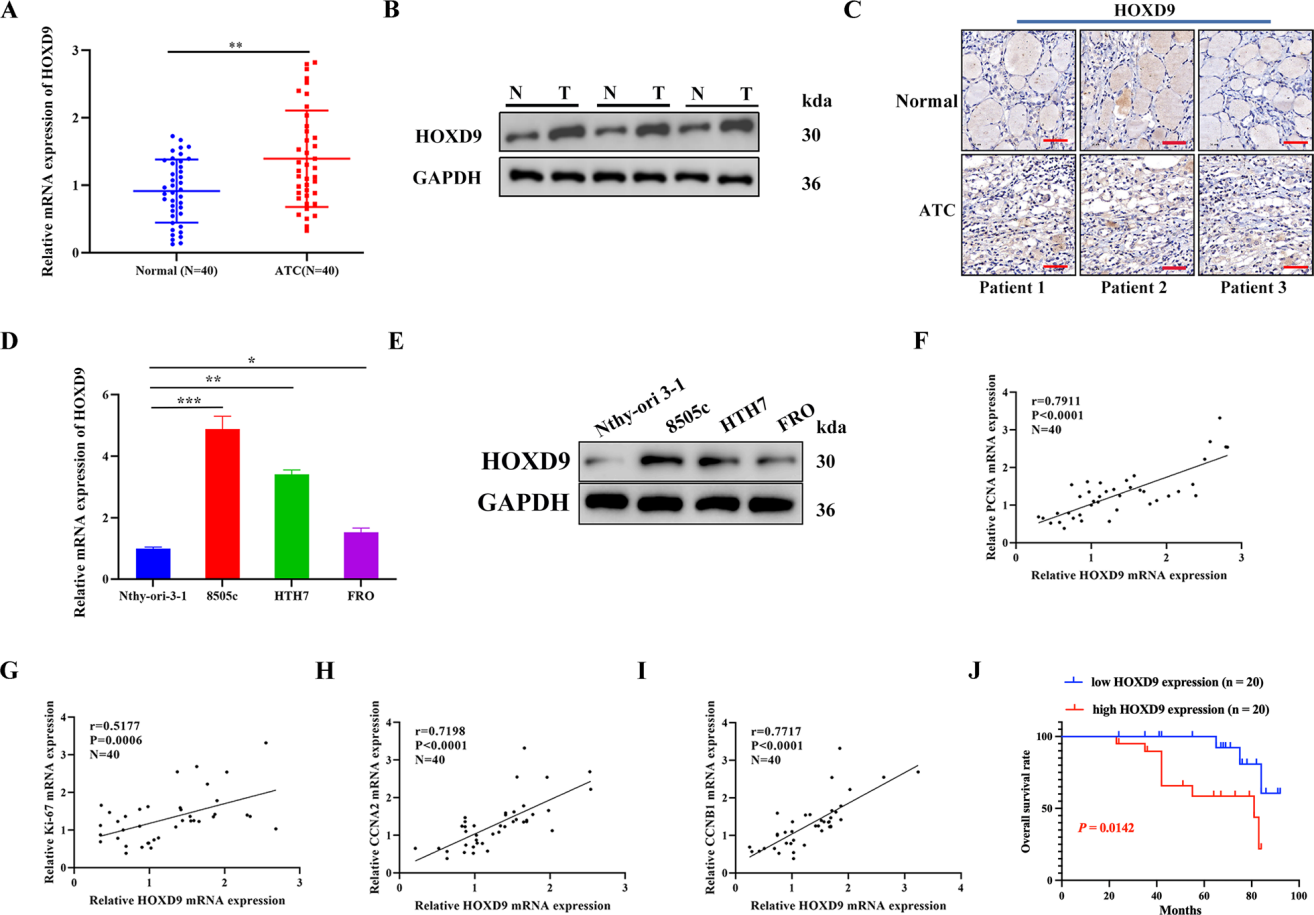
HOXD9 is upregulated in ATC tissues and correlates with a poor prognosis in patients. **A** qRT-PCR analysis showing the expression of HOXD9 in ATC tissues compared with matched normal para-cancerous tissues (n = 40). **B** HOXD9 protein expression was analyzed by western blotting in ATC tumor tissues and matched normal para-cancerous tissues (n = 3). **C** HOXD9 protein expression was analyzed by IHC analysis, scale bars: 50 μm. **D**, **E** HOXD9 expression in ATC cell lines 8505c, HTH7, FRO, and the normal thyroid cell line, Nthy-ori 3-1, assessed by qRT-PCR and western blotting analysis (n = 3). **F–I** *HOXD9* mRNA levels in ATC tissues positively correlate with cell proliferation markers (PCNA and Ki-67) and cell cycle-related genes (CCNA2, and CCNB1) in ATC patient tissues. **J** OS in 40 ATC patients is represented by Kaplan–Meier curves. The data represent mean values ± SD. *P < 0.05, **P < 0.01, ***P < 0.001

In this work, using pan-cancer perspective profiling datasets from The Cancer Genome Atlas (TCGA) database, we first examined HOXD9 expression in 24 tumor and normal tissues. The findings demonstrated that HOXD9 expression was elevated in most tumor tissues in comparison to normal tissues, and that the downregulation of HOXD9 expression in Thyroid Carcinoma (THCA) tissues was statistically significant (Additional file 1: Figure S1A). By accessing to the TCGA and Genotype-Tissue Expression, HOXD9 expression was found upregulated in THCA tissues (Additional file 1: Figure S1B). Subsequently, an examination was conducted to assess the expression of HOXD9 in various subtypes of THCA, including 11 cases of ATC, 49 cases of papillary thyroid carcinoma (PTC), and 45 cases of normal thyroid from the Gene Expression Omnibus (GEO) database (https://www.ncbi.nlm.nih.gov/geo/query/acc.cgi?acc = GSE33630) and discovered that HOXD9 expression is significantly higher in ATC and PTC than in the normal control group (Additional file 1: Figure S1C, D). In practical studies, we found that HOXD9 mRNA and protein expression was upregulated in the tissues of patients with ATC compared with matched adjacent noncancerous tissues (Fig. 1A, B). IHC analysis confirmed that HOXD9 expression was upregulated in the tissues of patients with ATC compared with matched adjacent noncancerous tissues (Fig. 1C). In addition, HOXD9 was also expressed significantly higher in ATC cell lines (8505c, HTH7, and FRO) than in a normal thyroid cell line (Nthy-ori 3–1), with the highest expression occurring in the 8505c cell line (Fig. 1D, E). As HOXD9 is an important transcription factor promoting tumor proliferation, we examined the correlation between *HOXD9* mRNA levels and proliferation markers PCNA, Ki-67 as well as cell cycle related genes CCNA2 and CCNB1 to validate whether HOXD9 can facilitate ATC proliferation. In ATC, *HOXD9* mRNA levels were positively correlated with PCNA (P < 0.001, r = 0.7911), Ki-67 (P < 0.001, r = 0.5177), CCNA2 (P < 0.001, r = 0.7198) and CCNB1 (P < 0.001, r = 0.7717). This indicates that the expression of HOXD9 is positively associated with tumor proliferation (Fig. 1F–I). Kaplan–Meier survival analysis performed on the TCGA-THCA datasets suggested that HOXD9 expression is not significantly associated with worse overall survival (OS) (log-rank test, P = 0.928; Additional file 1: Figure S1E). 40 ATC patients in the cohort were divided into two groups (high expression and low expression) according to the median *HOXD9* mRNA expression. The OS of patients analyzed by Kaplan–Meier curve demonstrated that high HOXD9 expression is significantly correlated with a poorer prognosis in ATC patients (Fig. 1J). Logistic regression analysis was used to evaluate the correlation between HOXD9 expression and clinical pathological characteristics, the results showed that higher expression of HOXD9 were positively correlated with tumor size, distant metastasis, and TNM stage, but not significantly with lymph node metastasis, gender, and age (Table 2**)**. Based on the results presented, we can conclude that HOXD9 plays an oncogenic role in THCA, particularly ATC. While HOXD9 is overexpressed in both PTC and ATC compared to normal thyroid tissues, its expression is significantly higher in ATC. Elevated HOXD9 levels strongly correlate with poorer OS in ATC patients, indicating its utility as a prognostic biomarker. Targeting HOXD9 may offer a novel therapeutic approach for this aggressive thyroid malignancy.

**Table 2:** Correlations between HOXD9 protein expression with clinical features in 40 ATC patients

Variables	Cases	**HOXD9**		χ^2^ value	*P* value
		Low (n = 20)	High (n = 20)		
Sex				1.9048	0.1675
Men	28	12	16		
Women	12	8	4		
Ages				0.1023	0.7491
< 60	17	8	9		
≥ 60	23	12	11		
Primary tumor size				3.9560	0.0467^*^
< 6 cm	14	4	10		
≥ 6 cm	26	16	10		
Lymph node metastasis				0.4167	0.5186
N0	16	7	9		
N1	24	13	11		
Distant metastasis				4.2857	0.0384^*^
M0	28	17	11		
M1	12	3	9		
Stage (AJCC, 2010)				8.19	0.0167^*^
IVA	14	10	4		
IVB	12	2	10		
IVC	14	8	6		

The median value of *HOXD9* mRNA expression was used as the cutoff for dividing patients into a high-expression group (n = 20) and a low-expression group (n = 20). P value when expression levels were compared using the Pearson Chi-square test. Bold values have statistical significance. *P < 0.05

### HOXD9 differential expression in the proliferation and apoptosis of ATC cells

**Fig. 2:**
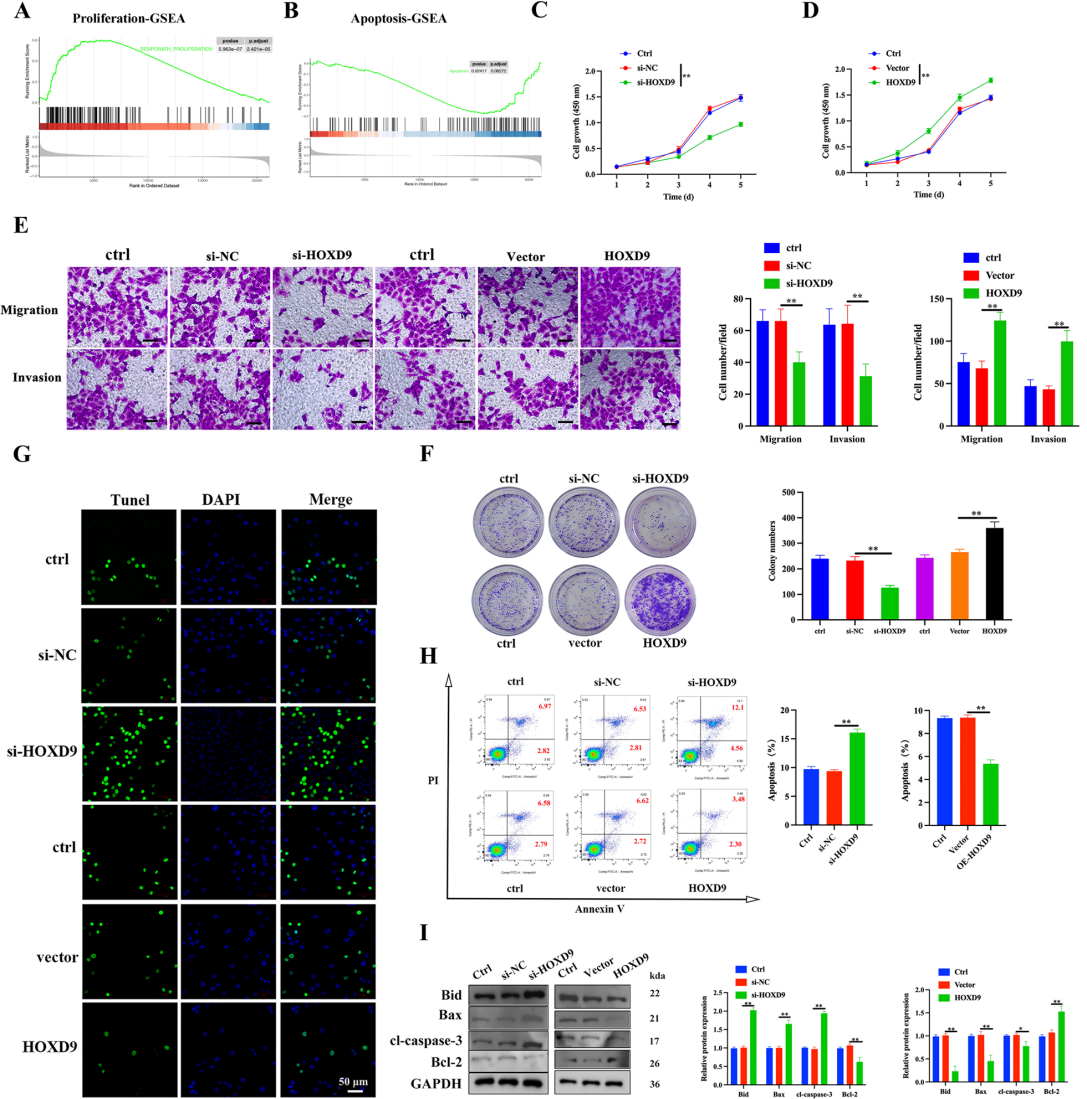
Effect of HOXD9 on 8505c cell proliferation and apoptosis. **A**, **B** GSEA analysis was performed using GESA software (v4.1.0) (http://www.gsea-msigdb.org/gsea/index.jsp) with gene sets database from GEO dataset (GSE76039). GSEA diagram was used to analyze the correlation between HOXD9 expression and proliferation/apoptosis signaling in ATC. **C**, **D** The cell growth of 8505c with HOXD9 silence or overexpression were assessed by CCK-8 assay. **E** Cell migration and invasion were assessed by Transwell assays, scale bar = 200 μm. **F** Tumor cell-induced colony formation was also analyzed, and the colony formation rate was calculated. **G** Cell apoptosis was examined by TUNEL in 8505c cells, scale bar, 50 μm. **H** Flow cytometry analysis of apoptosis in 8505c cells based on AnnexinV-FITC/PI. **I** Western blot analyses of apoptosis-related protein (Bid, Bax, cl-caspase-3, and Bcl-2) expression in 8505c cells. The data represent mean values ± SD, n = 3. *p < 0.05, **p < 0.01

Prior research has demonstrated that HOXD9 has the capacity to augment the proliferation of lung cancer, glioma, and other cellular entities, while concurrently impeding the tumor cell apoptosis [9, 26, 27]. However, the functional impacts of HOXD9 on the behaviors of ATC cells remain undetermined. Through the examination of the GEO dataset GSE76039, which encompasses information pertaining to 20 patients diagnosed with ATC and 17 patients diagnosed with poorly differentiated thyroid carcinoma, gene set enrichment analysis (GSEA) analysis was performed on the differentially expressed genes from RNA-sequence data, the enrichment plots results showed that a higher level of HOXD9 in ATC patients is positively associated with the enrichment of upregulation of proliferation and downregulation of apoptosis (Fig. 2A, B). To understand the exact involvement of HOXD9 in ATC proliferation and apoptosis, we knocked down and overexpressed HOXD9 in 8505c cells. The efficiency of interference and overexpression of HOXD9 in 8505c cells was verified by qRT-PCR and western blot analysis. Among them, si-HOXD9-3 had the best interference efficiency and was used as the subsequent experiment and named si-HOXD9 (Additional file 1: Figure S2A–E). CCK-8 and colony formation assays were performed to examine cell proliferation. HOXD9-silenced 8505c cells showed decreased cell viability in CCK-8 assay and formed fewer and smaller colonies in colony formation assays compared to the si-NC group, indicating impaired proliferation capacity when HOXD9 was knocked down (Fig. 2C, D, F). Transwell migration and invasion assays were carried out to determine cell motility. Knockdown of HOXD9 significantly inhibited the migration and invasion abilities of 8505c cells, as quantified by decreased number of cells passed through the Transwell chamber membrane (Fig. 2E). To assess apoptosis, TUNEL staining and flow cytometry analysis of Annexin V/PI stained cells were performed. A higher percentage of TUNEL-positive apoptotic cells and elevated Annexin V-stained cell population were observed in HOXD9-knockdown 8505c cells compared to the si-NC group, demonstrating induced apoptosis when HOXD9 was silenced (Fig. 2G, H). Western blotting further demonstrated altered expression of apoptosis-related proteins. Specifically, cleaved caspase-3, Bid and Bax were increased, while anti-apoptotic Bcl-2 was decreased in HOXD9-knockdown cells compared to the si-NC group. In contrast, overexpression of HOXD9 led to opposite effects in 8505c cells, including increased capabilities of proliferation, migration, invasion and decreased apoptosis (Fig. 2I). In summary, knockdown of HOXD9 facilitates proliferation, migration, invasion capacities and also induces apoptosis in 8505c cells, suggesting a tumor suppressive role.

### *Oncogenic activity of HOXD9 is *via* EMT in 8505c cells*

**Fig. 3:**
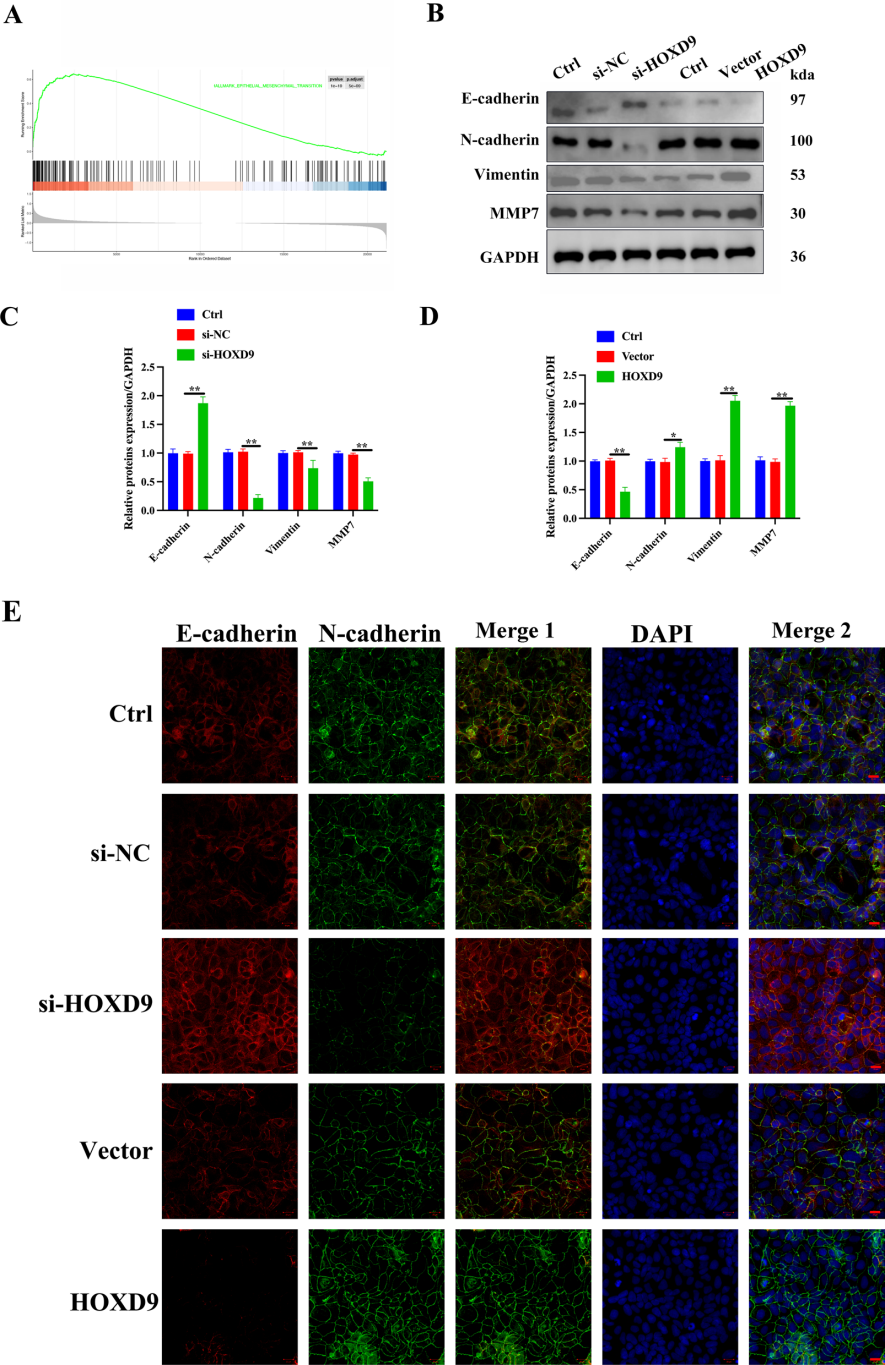
Oncogenic activity of HOXD9 is via EMT in 8505c cells. **A** GSEA indicated that HOXD9 was positively correlated with a functional gene cluster of EMT in ATC with gene sets database from GEO dataset (GSE76039). **B**–**D** The expression of EMT-related proteins was detected through western blot analysis. **E** Immunofluorescence was used to detect the expression of EMT-related proteins in 8505c cells, scale bar, 20 μm. The data represent mean values ± SD, n = 3. * P < 0.05, ** P < 0.01

GSEA indicated that HOXD9 was positively correlated with a functional gene cluster of EMT in ATC with gene sets database from GEO dataset (GSE76039) (Fig. 3A). Western blot analysis of a cell invasion marker (MMP7), epithelial marker (E-cadherin), and mesenchymal markers (Vimentin and N-cadherin) in the HOXD9 knockdown or overexpressing cells were compared with control groups. Knockdown of HOXD9 significantly increased the protein expression of E-cadherin, and decreased the protein expression of Vimentin, N-cadherin, and MMP7, whereas overexpression of HOXD9 decreased E-cadherin expression, but increased Vimentin, N-cadherin, and MMP7 protein expression (Fig. 3B–D). Moreover, we used immunofluorescence to detect the expression levels of E-cadherin and N-cadherin in 8505c cells with stable overexpression of HOXD9 or HOXD9 knockdown, and the results were consistent with the findings obtained by western blotting (Fig. 3E), indicating that HOXD9 promotes EMT in 8505c cells.

### HOXD9 promotes proliferation and metastasis by negatively regulating miR-451a in vitro

**Fig. 4:**
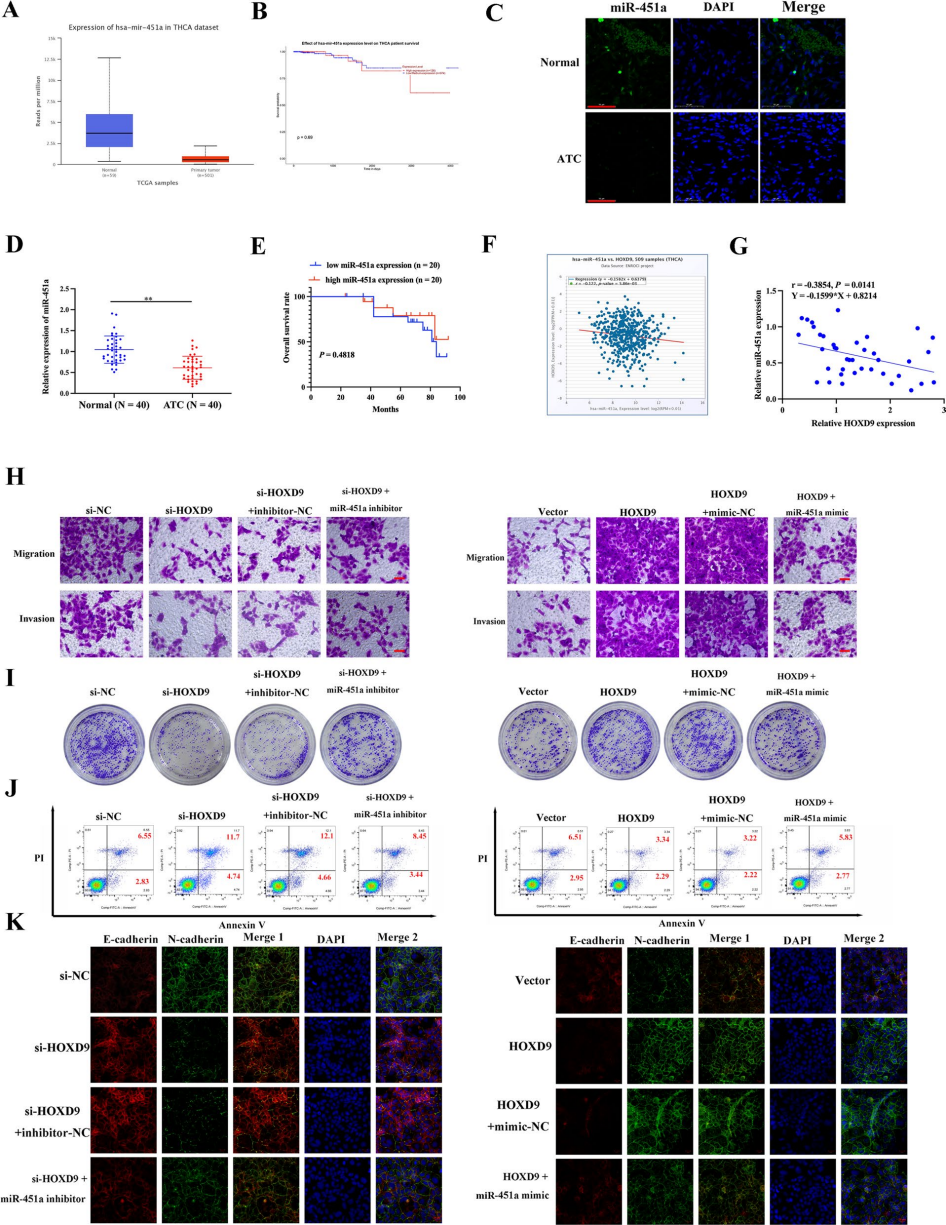
HOXD9 promotes proliferation and metastasis by regulating miR-451a. **A** TCGA cohort analysis of miR-451a expression level in pair-matched THCA samples. **B** Kaplan–Meier survival analysis for three miR-451a of THCA patients from TCGA. **C** Quantitative RT-PCR analysis of miR-451a levels in 40 pairs of ATC tissue and noncancerous tissue samples, U6 was used as the internal control. **D** Representative images of the expression of miR-451a in paired tissues using in situ hybridization. Scale bars: 50 μm. **E** Effects of miR-451a on prognosis and survival of 40 ATC patients. **F**, **G** Correlation analysis of miR-451a and *HOXD9* mRNA expression in ATC were analyzed by Pearson correlation analysis and "miRNA-Target CoExpression" module analysis in the starbase database. **H** Cell migration and invasion were measured by Transwell assays, scale bar, 50 μm. **I** Cell proliferation evaluated by colony formation assay. **J** Apoptosis of 8505c cells analyzed by flow cytometry. **K** The expression of E-cadherin and N-cadherin was detected through conducting immunofluorescence analysis, scale bar = 50 μm. The data represent mean values ± SD, n = 3. **P < 0.01

A previous study indicated that miR-451a could inhibit cancer growth and EMT and induce apoptosis in PTC [28]. The analysis of the THCA dataset from the TCGA database shows that the expression of miR-451a is low in THCA (Fig. 4A). However, the low expression of miR-451a was not correlated with a lower patient survival rate (Fig. 4B). We next explored the expression of miR-451a in ATC. The results of qRT-PCR and in situ hybridization showed that the relative expression of miR-451a was lower in ATC tissues than in normal tissues (Fig. 4C, D). The miR-451a expression levels were classified as either low or high according to the median of the cohort. Our data provide evidence that low expression of miR-451a was not correlated with a lower patient survival rate in ATC (Fig. 4E). By combining UCSC and JASPAR, we identified HOXD9 as an upstream regulator of miR-451a. Correlation analysis of miR-451a and *HOXD9* mRNA expression in ATC tumor tissues of 40 patients were analyzed by Pearson correlation and the "miRNA-Target CoExpression" module analysis in starBase (https://starbase.sysu.edu.cn/panMirCoExp.php) (Fig. 4F, G). The results hinted that HOXD9 and miR-451a may have a negative feedback regulation in ATC. The association between miR-451a expression and the clinicopathological features of ATC was assessed in 40 ATC patient samples, high or low expression of miR-451a was not correlated to clinicopathological features (Table 3). To further investigate the relationship between HOXD9 and miR-451a we conducted a series of experiments with HOXD9-knockdown, miR-451a inhibitor, HOXD9-overexpression, and miR-451a mimic transfected into 8505c cells. We investigated the effect of HOXD9 on 8505c cell progression by controlling miR-451a. A CCK-8 assay showed that HOXD9 interference inhibited the propagation of 8505c cells, however, the miR-451a inhibitor restored the impaired effect induced by silencing HOXD9 (Additional file 1: Figure S3A). In addition, miR-451a mimic treatment could counteract the elevated cell viability caused by HOXD9 overexpression (Additional file 1: Figure S3B). Furthermore, as a result of HOXD9 interference the migration and invasion capacity of 8505c cell lines were inhibited, whereas when miR-451a was inhibited, the migration and invasion capacity of the cells were partially restored. Similarly, HOXD9 overexpression increased the invasion and migration of 8505c cells, whereas the miR-451a mimic reversed this effect (Fig. 4H, Additional file 1: Figure S3C, D). Moreover, treatment with miR-451a inhibitor and miR-451a mimic restored the reduction and increase in proliferation of 8505c cells caused by interference and overexpression of HOXD9, respectively (Fig. 4I, Additional file 1: Figure S3E, F). Flow cytometry results show that miR-451a inhibitor and miR-451a mimic counteract the increased and decreased levels of apoptosis in ATC cells due to HOXD9 interference or overexpression, respectively (Fig. 4J, Additional file 1: Figure S3G, H). EMT characteristics and apoptosis were also determined in cells with HOXD9 overexpressed and transfected with miR-451a mimic or in cells with HOXD9 silenced and transfected with a miR-451a inhibitor (Fig. 4K, Additional file 1: Figure S3I, J). The results indicate that HOXD9 promotes proliferation and metastasis by negatively regulating miR-451a in ATC.

**Table 3:** Correlations between miR-451a expression with clinical features in 40 ATC patients

Variables	Cases	**miR-451a**		χ^2^ value	*P* value
		Low (n = 20)	High (n = 20)		
Sex				1.905	0.166
Men	28	12	16		
Women	12	8	4		
Ages				0.1023	0.7491
< 60	17	9	8		
≥ 60	23	11	12		
Primary tumor size				0.4396	0.5073
< 6 cm	14	8	6		
≥ 6 cm	26	12	14		
Lymph node metastasis				0.6455	0.5186
N0	16	9	7		
N1	24	11	13		
Distant metastasis				0.4762	0.4902
M0	28	13	15		
M1	12	7	5		
Stage (AJCC, 2010)				2.340	0.3104
IVA	14	9	5		
IVB	12	6	7		
IVC	14	5	9		

The median value of miR-451a expression was used as the cutoff for dividing patients into a high-expression group (n = 20) and a low-expression group (n = 20). P value when expression levels were compared using the Pearson Chi-square test

### Transcription factor HOXD9 negatively regulates miR-451a by directly binding to its promoter region

**Fig. 5:**
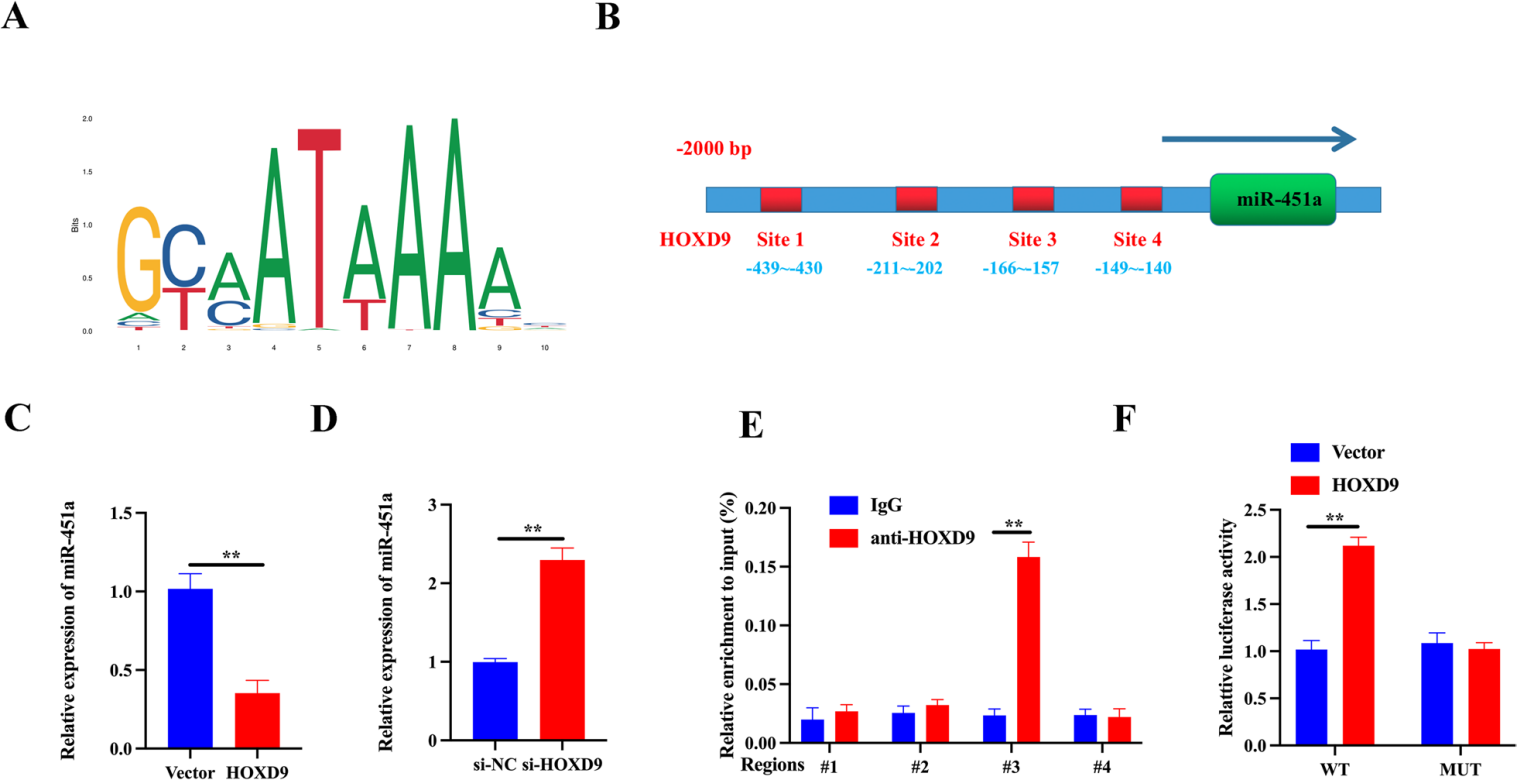
Transcription factor HOXD9 negatively regulates miR-451a by directly binding to its promoter region. **A** Binding motif of HOXD9 in the promoter of miR-451a, predicted by JASPAR (http://jaspar.genereg.net/). **B** JASPAR predicted the potential binding sites for HOXD9 in the miR-451a promotor. **C**, **D** The expression of HOXD9 was overexpressed and suppressed in 8505c cells, and miR-451a expression was identified by qRT-PCR; U6 was used as the internal control. **E** Result of ChIP assay evaluating the enrichment of HOXD9 at the promoter region of miR-451a. **F** Relative luciferase activities in 8505c cells transfected with a HOXD9 overexpression plasmid. The data represent mean values ± SD. **P < 0.01

To verify whether HOXD9 directly regulated miR-451a expression through transcription, we analyzed the potential binding sites of HOXD9 within the 2,000 bp 5′-promoter region of miR-451a through using the Ensembl and PROMO 3.0 websites. By searching the JASPAR database, we found the binding motifs of HOXD9 and four potential HOXD9 binding sites on the miR-451a promoter (Fig. 5A, B). qRT-PCR analysis demonstrated that overexpression of HOXD9 in 8505c cells significantly decreased miR-451a levels, whereas HOXD9 deletion had the opposite effect (Fig. 5C, D). ChIP-qPCR results indicated that HOXD9 could interact with miR-451a promoter within the − 166 to − 157 bp region (BS 3) (Fig. 5E). In addition, dual luciferase reporter assay showed that the luciferase activity of miR-451a promoter-WT was decreased following OE-HOXD9 transfection whereas no statistical changes were observed in the luciferase activity of miR-451a promoter-MUT (Fig. 5F).

### PSMB8 is a direct target of miR-451a and has a pro-carcinogenic role in ATC

**Fig. 6:**
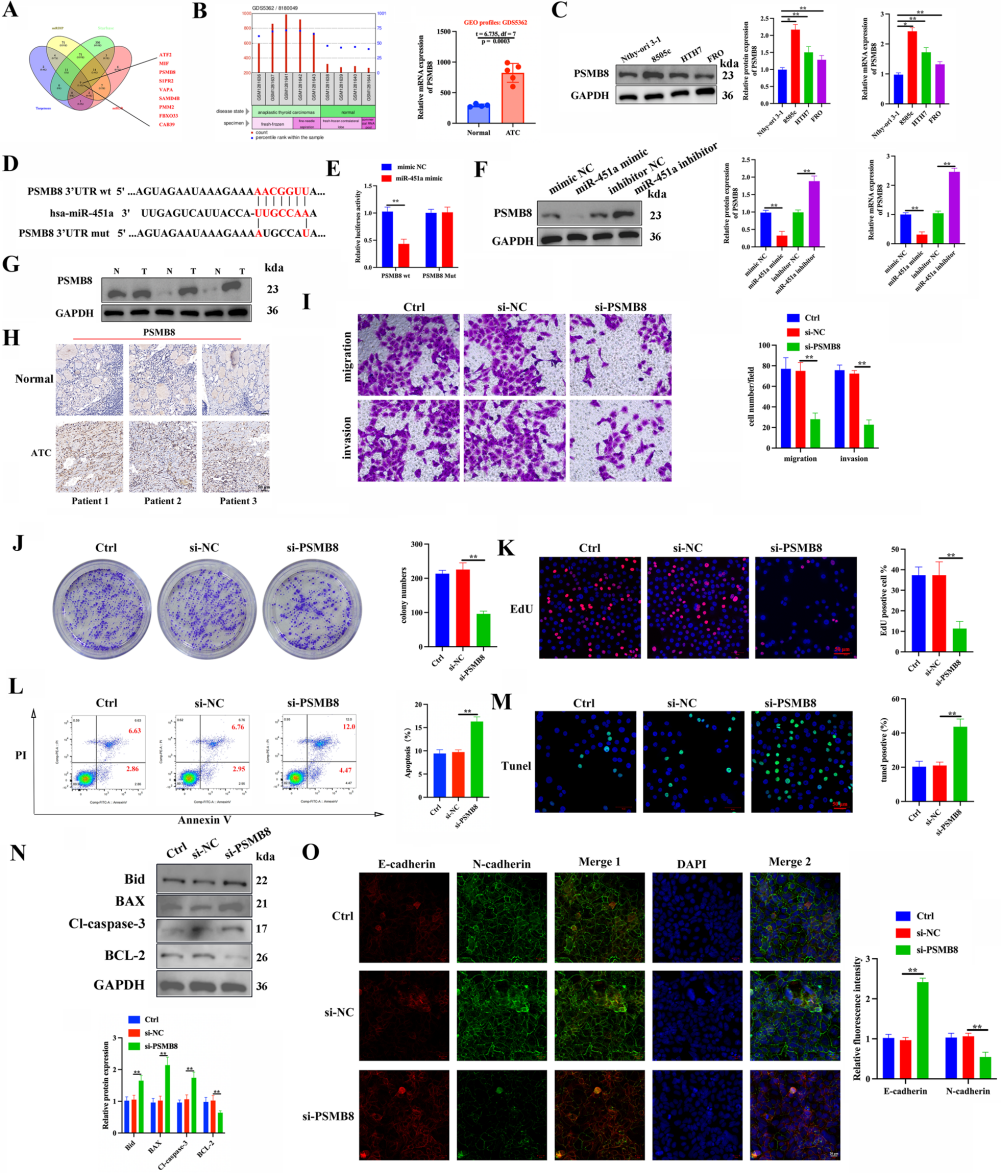
PSMB8 is a direct target of miR-451a and has a pro-carcinogenic role in ATC. **A** Online target gene prediction tools (TargetScan, miRDB, mirDIP, and starBase) predicted that miR-451a had nine shared target genes. **B** PSMB8 mRNA expression acquired from GEO profiles database (GDS5362) which contain five ATC tissues and four normal thyroid tissues. **C** PSMB8 mRNA and protein expression in ATC cell lines 8505c, HTH7, FRO, and the normal thyroid cell line, Nthy-ori 3–1, assessed by RT-PCR and western blot analysis. **D** Sequence alignment of predicted miR-451a BS within the 3′UTR of *PSMB8* mRNA. **E** 8505c cells transfected with a luciferase reporter of the wildtype or a mutant 3′UTR of *PSMB8* mRNA were co-transfected with miR-451a, miR-NC, inhibitor-451a, or inhibitor-NC. Firefly luciferase activity was detected and normalized by Renilla luciferase activity. **F** *PSMB8* mRNA and protein expression in ATC cell lines 8505c, HTH7, FRO, and the normal thyroid cell line, Nthy-ori 3–1, assessed by RT-PCR and western blot analysis. **G** Relative protein expression levels of PSMB8 in ATC tumor tissues and non-tumor adjacent tissues was compared by western blotting analysis, N, normal tissue, T, tumor tissue. **H** PSMB8 protein expression was analyzed by IHC in ATC tissues, scale bars: 50 μm. **I** Transwell migration and invasion assays were performed in 8505c cells after transfection with si-NC and si-PSMB8. **J** The tumor cell 8505c-induced colony formation was also analyzed, and the colony formation rate was calculated. **K** EdU staining for proliferation analysis, scale bars: 50 μm. **L**, **M** Flow cytometry and TUNEL staining analysis of apoptosis in 8505c cells based on AnnexinV-FITC/PI, scale bars: 50 μm. **N** Western blotting analysis of apoptosis-related proteins. **O** The expression of epithelial–mesenchymal transition-related proteins were detected through conducting immunofluorescence, scale bar = 20 μm. The data represent mean values ± SD, n = 3, *P < 0.05, **P < 0.01

Using online target gene prediction tools (TargetScan, miRDB, mirDIP, and starBase), we were able to predict nine shared target genes of miR-451a (Fig. 6A). Correlation between HOXD9 and miR-451a target genes in thyroid cancer was analyzed on the ENCORI pan-cancer analysis platform (http://starbase.sysu.edu.cn/panCancer.php) using the expression data from TCGA. Among the nine miR-451a downstream target genes that were determined to be shared, we discovered that PSMB8 was the only one of the above nine genes that was highly expressed in THCA, which may be positively correlated with HOXD9 and negatively correlated with miR-451a (Additional file 1: Figure S4A–D). In addition, *PSMB8* mRNA levels were significantly up-regulated in ATC tissues when compared to normal thyroid tissues from searching the GEO profiles database (GDS5362) (Fig. 6B). Results of qRT-PCR and western blotting analysis showed that PSMB8 expression was highest in 8505c cells compared to the normal thyroid cell line, Nthy-ori 3–1 (Fig. 6C). In view of the specificity and relatively low incidence of ATC among THCA subtypes, we chose ATC cell lines to perform the subsequent dual-luciferase reporter assay to determine the regulatory relationship between miR-451a and PSMB8 in ATC. The dual-luciferase reporter assay can validate the direct interaction between miR-451a and PSMB8 in a relatively isolated condition. The predicted miR-451a binding site in the 3′UTR of PSMB8 mRNA was mutated (Fig. 6D). Luciferase activity confirmed an interaction between PSMB8 and miR-451a (Fig. 6E). The expression of PSMB8 mRNA and protein levels in 8505c cells were lower when miR-451a was overexpressed than when it was suppressed (Fig. 6F). We next explored whether PSMB8 plays a similar role to HOXD9 as a pro-oncogene in ATC. First, we examined PSMB8 protein expression in clinical ATC tissue samples to determine the role of PSMB8 in the development of ATC. The western blotting and IHC analysis results showed that the protein expression of PSMB8 was significantly higher in ATC tumor tissues than in non-tumor adjacent tissues (Fig. 6G, H). The association between PSMB8 protein expression levels and clinicopathological features of 40 ATC patients is shown in Table 4. We clearly demonstrate that the high expression of PSMB8 significantly correlates with distant metastasis in ATC tissues.

**Table 4:** Correlations between PSMB8 protein expression with clinical features in 40 ATC patients

Variables	Cases	**PSMB8**		χ^2^ value	*P* value
		Low (n = 20)	High (n = 20)		
Sex				3.2516	0.0714
Men	28	12	16		
Women	12	8	4		
Ages				0.9207	0.3373
< 60	17	10	7		
≥ 60	23	10	13		
Primary tumor size				0.6205	0.4309
< 6 cm	14	8	6		
≥ 6 cm	26	12	14		
Lymph node metastasis				0.4167	0.5186
N0	16	9	7		
N1	24	11	13		
Distant metastasis				4.286	0.0384^*^
M0	28	17	11		
M1	12	3	9		
Stage (AJCC, 2010)				1.762	0.4144
IVA	14	9	5		
IVB	12	5	7		
IVC	14	6	8		

The median value of *PSMB8* mRNA expression was used as the cutoff for dividing patients into a high-expression group (n = 20) and a low-expression group (n = 20). P value when expression levels were compared using the Pearson Chi-square test. Bold values have statistical significance. *P < 0.05

After evaluating the PSMB8 silencing efficiency in 8505c cells through qRT-PCR and western blotting (Additional file 1: Figure S4E–G), we chose the most effective silencing efficiency to continue. Following this, we examined the effect of PSMB8 on ATC cells in vitro to evaluate whether it had the same effect as HOXD9 in promoting the development of ATC. First, we used a Transwell assay to investigate cell migration and invasion, and then we used a clone formation assay and EdU staining to examine cell proliferation. When PSMB8 is knocked down in 8505c cells, cell migration, invasion, and proliferation are reduced (Fig. 6I–K). The key distinction was that interfering with PSMB8 led to an increase in the rate of apoptosis (Fig. 6L–N). Similarly, interfering with PSMB8 was able to stop the advancement of EMT (Fig. 6O). In summary, PSMB8 plays a similar role to HOXD9 in ATC as an oncogenic factor.

### Activation of PI3K/Akt pathway is essential for induction of HOXD9/miR-451a/PSMB8 axis-mediated ATC malignant progression

**Fig. 7:**
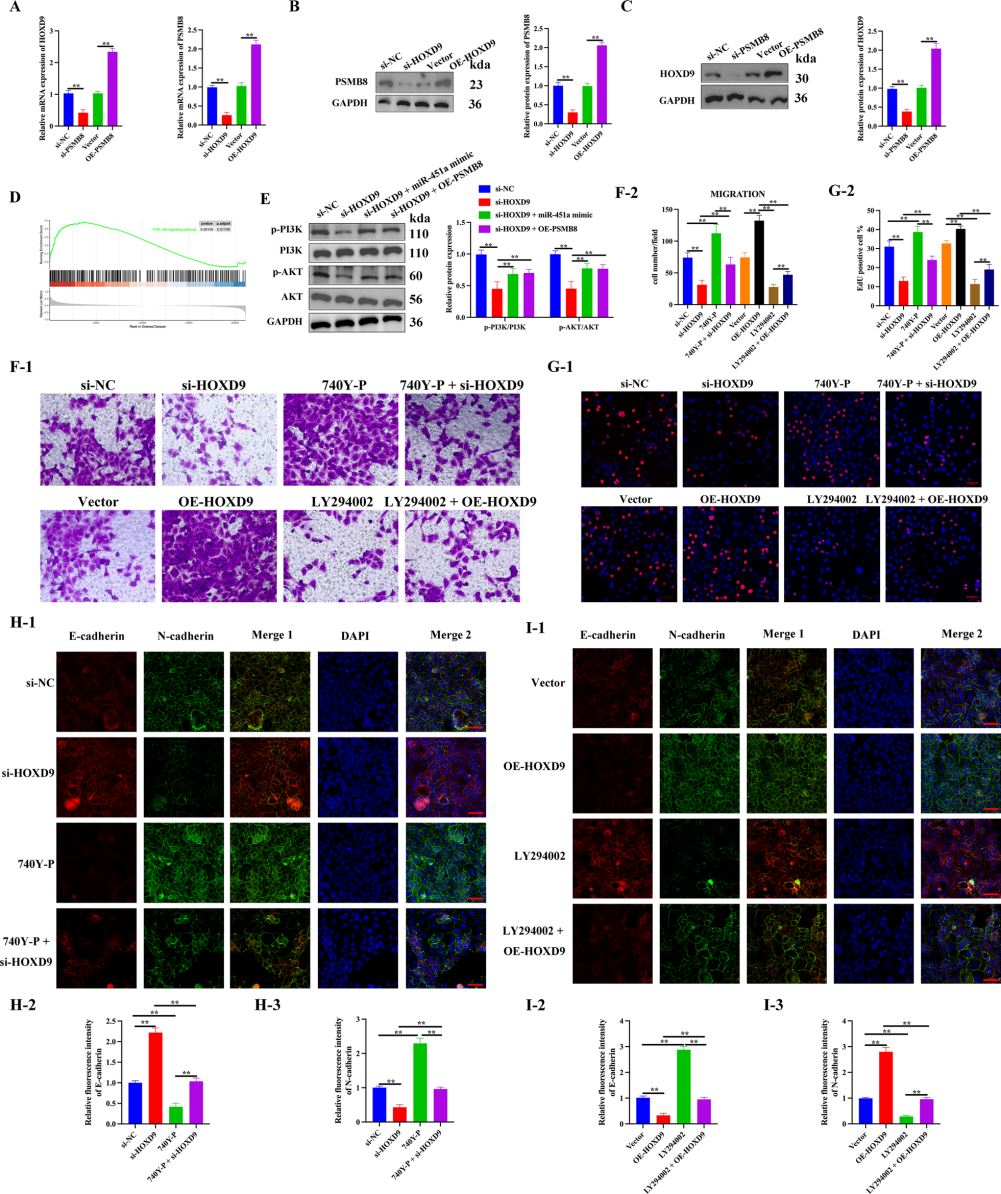
Activation of the PI3K/Akt pathway is essential for the induction of HOXD9/miR-451a/PSMB8 axis-mediated ATC malignant progression. **A–C** Detection of HOXD9 and PSMB8 expression in the presence or absence of PSMB8 by qPCR and western blotting. **D** GSEA diagram was used to analyze the correlation between HOXD9 expression and PI3K/AKT signaling in GEO dataset (GSE76039). **E** PI3K/AKT signaling pathway proteins were measured by western blotting. **F** Transwell migration assays. **G** Cell proliferation was detected by EdU staining, scale bar = 50 μm. **H**, **I** The expression of EMT-related proteins were detected by immunofluorescence, scale bar = 50 μm. The data represent mean values ± SD, n = 3, **P < 0.01

Results by qRT-PCR and western blot analysis indicated that the interference and overexpression of HOXD9 in 8505c cells resulted in decreased and increased expression of PSMB8. Similarly, the interference and overexpression of PSMB8 also resulted in decreased and increased expression of HOXD9, indicating a positive regulatory link between the two genes (Fig. 7A–C). Through the regulation of downstream components, activation of the PI3K/AKT pathway is known to be associated with cancer cell proliferation, survival, and apoptosis [29]. Besides, a GSEA plot showing that HOXD9 expression was positively correlated with PI3K/AKT signaling pathway in the GEO dataset (GSE76039) (Fig. 7D). Therefore, we next explored whether HOXD9/miR-451a/PSMB8 axis could promote ATC malignant progression via activating the downstream PI3K/AKT signaling pathway. Silencing HOXD9 in 8505c cells leads to inhibition of PI3K/AKT pathway activation, whereas miR-451a mimic or the overexpression of PSMB8 can mitigate the effect of HOXD9 interference (Fig. 7E). To determine whether the effect of HOXD9 on ATC carcinogenesis is mediated through the PI3K/AKT pathway, PI3K activator 740Y-P (25 μM) and inhibitor LY294002 (30 μM) were used to inhibit or activate the PI3k/AKT pathway. Next, we examined the changes in migration, proliferation, and EMT levels of 8505c cells after inhibition or activation of the PI3K/AKT pathway in the presence of overexpressed or disrupted HOXD9. According to the findings, blocking the PI3K/AKT pathway caused ATC cells to migrate, proliferate, and undergo EMT less frequently than when the system was left active. The blocking or activation of the PI3K/AKT pathway attenuates the effect of si-HOXD9 or OE-HOXD9 on ATC progression. Overall, these results demonstrated that the PI3K/AKT signaling pathway was involved in HOXD9-stimulated ATC cell proliferation and EMT (Fig. 7F-I).

### Silencing HOXD9 attenuates tumor growth in vivo

**Fig. 8:**
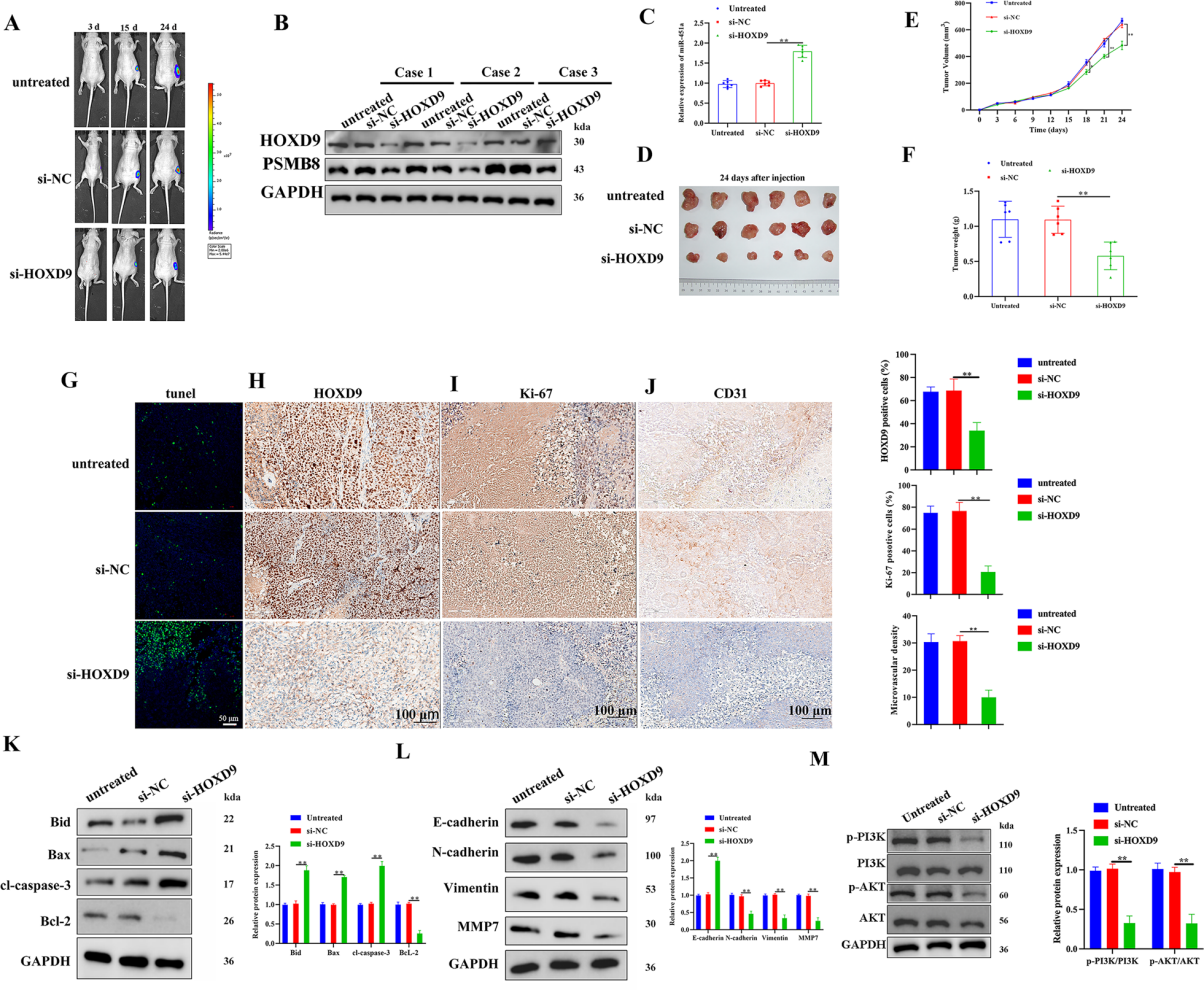
Silencing HOXD9 attenuates tumor growth in vivo. **A** Tumor growth progression was measured by i*n vivo* luciferase imaging of the xenografts at days 3, 15, and 24 after inoculation. **B** The expression of HOXD9 and PSMB8 in tumors of mice was estimated by western blotting, n = 3. **C** The expression of miR-451a in tumors of mice was estimated by RT-PCR, with U6 as a loading control, n = 6. **D** Representative gross photos of tumors 28 d after subcutaneous xenografting, n = 6. **E** Tumor growth curves were monitored and calculated after 0, 3, 6, 9, 12, 15, 18, 21, and 24 d of inoculation. **F** Body weight changes in the three groups were monitored 24 day after subcutaneous xenografting. **G**–**J** Fluorescence TUNEL assay was carried out to determine cell apoptosis in the same tumor tissues as indicated above. The protein levels of HOXD9, Ki-67, and CD31 in tumors were estimated by IHC analysis. **K**–**M** Representative results of western blot analyses of apoptosis, EMT-related, and PI3K/AKT pathway proteins in tumor tissues, with GAPDH as a loading control. The data represent mean values ± SD. **P < 0.01

We established an orthotopic mouse thyroid model using 8505c cells expressing si-HOXD9 to determine if HOXD9 silencing could affect tumor growth in vivo. After inoculation, in vivo luciferase imaging of the xenografts allowed for the measurement of tumor growth (Fig. 8A). The expression of HOXD9, PSMB8, and miR-451a was determined in excised tumors (Fig. 8B, C). As expected, protein levels of HOXD9 and PSMB8 were lower when HOXD9 was suppressed whereas levels of miR-451a were higher. Consequently, the knockdown of HOXD9 significantly suppressed the tumor growth, as revealed by the impaired increase of tumor volume on days 18, 21, and 24 after inoculation as well as the reduced tumor weight in the si-HOXD9 group (Fig. 8D–F). CD31, an endothelial cell marker used to assess tumor angiogenesis, promotes the development and spread of tumors [30]. The protein levels of HOXD9, Ki-67, and CD31 in tumors were estimated by IHC and a fluorescence TUNEL assay was used to determine cell apoptosis in the same tumor tissues (Fig. 8G–J). The apoptosis rate was significantly higher and cell proliferation and microvascular density were lower when HOXD9 is suppressed in tumor cells. This was confirmed by measuring protein levels of Bid, Bax, Bcl-2, and cl-caspase3 (Fig. 8K). There was also a significantly lower rate of EMT in tumors with HOXD9 silenced (Fig. 8L). The western blot results on tumor tissues revealed that silencing of HOXD9 blocked the PI3K/AKT signaling pathway (Fig. 8M). These results collectively indicate that suppressing the expression of HOXD9 can attenuate tumor growth in an orthotopic murine thyroid model of ATC.

### HOXD9 promotes metastasis in vivo

**Fig. 9:**
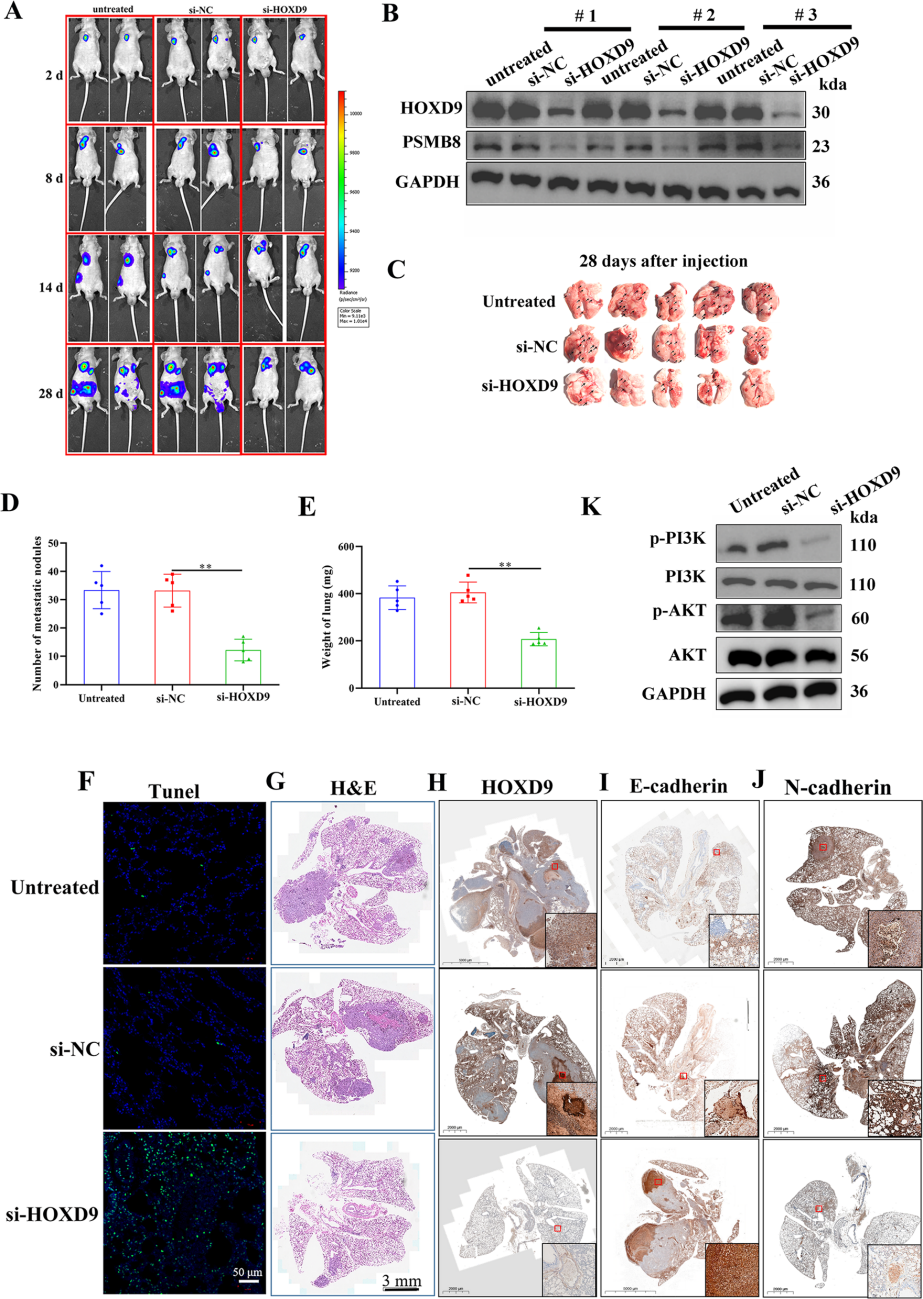
HOXD9 promotes metastasis in vivo. **A** In vivo luciferase imaging of the xenografts at days 2, 8, 14, and 28 following inoculation was used to track tumor development. The size of the tumor was determined by measuring the bioluminescent signal (photons/second/cm^2^/sr) in a ROI drawn around the tumor. **B** Representative results of western blot analyses of HOXD9 in lung tissues, GAPDH as a loading control. **C** The lungs were removed and imaged after 28 days of injection through the tail vein, n = 5. **D** The number of metastatic nodules in lungs was determined. **E** The weight of the lungs was calculated. **F** Fluorescence TUNEL assay was carried out to determine cell apoptosis in the lung tissues, scale bar, 50 μm. **G** H&E staining, scale bar, 3 mm. **H–J** IHC analysis of HOXD9, E-cadherin, and N-cadherin, scale bar, 500 μm. The data represent mean values ± SD. **P < 0.01

We also evaluated the level of tumorigenesis and metastasis in the orthotopic murine thyroid model with ATC cells containing si-HOXD9. Tumor size was determined by measuring the bioluminescent signal in a region of interest (ROI) drawn around the tumors at specific time points (2, 8, 14, and 28 d). Suppressing the expression of HOXD9 significantly reduced the size of tumors in vivo (Fig. 9A, B). Moreover, metastases measured by the number of metastatic nodules in excised lungs was significantly reduced when HOXD9 was silenced and the overall physiology of the lung improved (Fig. 9C–E). A fluorescence TUNEL assay indicated an improvement in apoptosis in lung tissue (Fig. 9F). Histological H&E staining of lung tissue sections showing metastasis has developed in mice injected with untreated or si-HOXD9 transduced 8505c cells (Fig. 9G). The ratio of HOXD9, E-cadherin, and N-cadherin in lung tissue measured by IHC analysis indicated that HOXD9 interference can prevent EMT in lung tissue (Fig. 9H–J). Similar to the results of the subcutaneous tumorigenesis assay, western blot analysis of lung tissue also showed that the silencing of HOXD9 blocked the PI3K/AKT signaling pathway (Fig. 9K). Overall, these results demonstrate that the silencing of HOXD9 can prevent lung metastases in an orthotopic murine thyroid model.

## Discussion

Although the rate of several cancers is in decline, the rate of thyroid cancer has tripled over the last decade and whereas differentiated cancers can be managed easily, the prognosis for undifferentiated thyroid cancers, such as ATC, remains dismal [31]. ATC is a particularly deadly malignant tumor that has a poor outlook due to a high degree of aggressiveness, and the potential mechanisms involved in ATC progression remain unclarified [32]. To make advances in the treatment of ATC, the primary obstacles are gaining an understanding of the novel mechanisms that underlie advancement and locating new targets that can prevent this progression. This implies that a greater understanding of the molecular progression of ATC is needed to develop more effective therapies. The HOXD9 transcription factor is associated with EMT in several malignancies [11, 18, 33]. However, the regulatory roles of HOXD9 in ATC are unclear. Consequently, the current work was designed to investigate the role of HOXD9 in the development of ATC and its molecular processes. We found that HOXD9 was typically overexpressed in ATC tissues, had a favorable correlation with clinicopathological characteristics (tumor size, distant metastasis, and TNM stage), and was an independent prognostic predictor for ATC patients. In addition, using a variety of in vitro and in vivo gain and loss of expression assays, we were able to demonstrate that HOXD9 has a functional role in the processes of cell proliferation, migration, invasion, apoptosis, and EMT in ATC. Specifically, we investigated ways in which HOXD9, miR-451a, and PSMB8 interact with one another in ATC.

According to recent studies, HOXD9 plays an important role in a variety of clinical processes and might be a valuable diagnostic and prognostic indicator for cancer patients. HOXD9 expression was greater in ATC tissues compared to matching normal non-cancerous tissues in our investigation, demonstrating its clinical importance. Some key clinicopathological features showed an association between high levels of HOXD9 and an increased risk of ATC progression, which suggests that HOXD9 may play an oncogenic role in the progression of the disease. Further research into this possibility would be extremely valuable for improving the treatment of ATC.

The aggressiveness of ATC and its high propensity for metastasis provide significant difficulties in its management [34]. In this particular study, we discovered that interfering with HOXD9 was related to a reduction in the migratory and invasive capabilities of ATC cells in vitro, as well as a lowering of cell survival. Inhibition of HOXD9 gene expression in vitro significantly decreased the proliferation, migration, and invasiveness of ATC cell lines. In parallel, we found that knocking down HOXD9 in vivo dramatically reduced tumor growth and lung metastasis development. Our results are consistent with the results of a previous study on cervical cancer [35].

EMT activation is a hallmark of the metastatic phase of cancer [36] and is a key component of cancer cell migration and invasion. As a result, cells that are normally epithelial become mesenchymal, and these cancer cells proliferate to generate new tumors [37]. In this study, we found that the knockdown of HOXD9 inhibited cell proliferation, migration, invasion, and EMT but increased apoptosis in ATC cells. The results we obtained by downregulating HOXD9 corresponded to those found in other studies [12, 35, 38, 39]. Chen et al. [39] found that *HOXD9* was part of a metastasis-related five-gene signature that could be used to predict survival in HCC. From 1895 metastasis-related genes, they found that the expression of SLC2A1, CDCA8, ATG10, and HOXD9 was higher in HCC tumor tissue whereas the expression of TPM1 was lower. In cervical cancer, HOXD9 was found to bind to the promoter of the P97 oncogene to regulate the expression of the human papillomavirus 16 E6/E7 genes and thereby promote survival, proliferation, and metastasis of cervical cancer cells [35].

By combining UCSC and JASPAR, we identified that HOXD9 was an upstream regulator of miR-451a. Several studies have discovered that interactions between HOXD9 and miRNA can influence tumor proliferation, metastasis, or EMT. Studies by Dai et al. and Lin et al. reported that miR-205 could inhibit EMT in glioma and breast cancer by downregulating HOXD9 [18, 40]. In addition, a study by Li et al. [41], found that the inhibition of miR-135a by lncRNA UCA1 could induce the activation of HOXD9 and promote the occurrence of EMT in glioma. In our study, we discovered that HOXD9 negatively regulates miR-451a by directly binding to its promoter region, HOXD9 promotes proliferation and metastasis by negatively regulating miR-451a in vitro.

In this study, we discovered that PSMB8 had a similar molecular profile to HOXD9 in ATC and they were both upregulated in tumor tissue. Strong expression of PSMB8 was also found to be correlated with distant metastasis in ATC. The knockdown of PSMB8 decreases HOXD9 expression and the suppression of HOXD9 consequently decreased the expression of PSMB8. PSMB8 is associated with several cancers as both a tumor promoter and suppressor. Suppression of PSMB8 may be a promising strategy for the treatment of PSMB8-overexpressing gliomas [23]. In contrast, PSMB8 was downregulated in gastric cancer tissue and was found to inhibit proliferation and promote apoptosis in cancer cells [24]. Novel potential targets of miR-451a have been found using online target gene prediction tools. PSMB8 was chosen as the most promising candidate among these potential targets. The prediction results using TargetScan were verified by a luciferase activity reporter experiment. In this study, we found that PSMB8 was targeted by miR-451a to promote ATC cell proliferation, similar results were found in prostate cancer [42].

The PI3K/AKT pathway has been confirmed to play a key role in dedifferentiation and tumor cell growth in ATC [29]. Previous research suggested that HOXD9 could influence the progression of osteosarcoma malignancy via the PI3K/AKT/mTOR pathway [43]. Through the modulation of the PI3K/AKT/mTOR pathway, miR-451a has the potential to operate as a tumor suppressor in patients with gastric cancer [44]. PSMB8 has also been demonstrated to influence glioma cell migration, proliferation, and apoptosis via modifying the PI3K/AKT signaling pathway [23]. In the current investigation, we concluded that the activation of the PI3K/AKT pathway is essential for the HOXD9/miR-451a/PSMB8 axis-mediated induction of ATC malignant development.

This study provides novel insights into the tumor-promoting role of HOXD9 in ATC. Although its functional role in cancer development and progression remains unclear, the differential expression of these proteins between tumor and normal cells makes them prime candidates for cancer targeted therapy. However, therapeutic inhibition of HOXD9 may therefore have unintended impacts on normal physiological processes. It will be critical to evaluate the effects of anti-HOXD9 therapies on normal cells, such as through toxicity studies in non-tumorigenic cell lines. Strategies to enhance the selectivity of HOXD9 targeting include the development of inhibitors against mutated forms of the protein found specifically in cancer cells. Delivery methods that target anti-HOXD9 agents preferentially to tumor sites could also reduce off-target effects. Furthermore, intermittent dosing regimens may balance efficacy against undesirable impacts on normal tissue homeostasis. As HOXD9-targeted therapies are explored, thorough investigations of their selectivity profiles will be essential to maximize anticancer effects while minimizing risks to normal tissue function.

There are several limitations that could be addressed in future investigations. First, the sample size of clinical ATC tissues was relatively small. Expanding the cohort across multiple centers could improve generalizability of the findings. Second, the in vivo experiments focused solely on HOXD9 knockdown, without assessing impacts of HOXD9 overexpression. Future animal studies evaluating both gain- and loss-of-function models could help elucidate the mechanisms of HOXD9-mediated ATC progression more comprehensively. Third, additional bioinformatic approaches like transcriptomic profiling or single-cell RNA sequencing of 8505c cells could be leveraged to identify other key ATC regulators beyond the HOXD9/miR-451a/PSMB8 axis. Moving forward, integrating multi-omics datasets with larger clinical cohorts and more diverse preclinical models will be imperative to fully define the complex signaling networks driving aggressive behaviors in ATC. Targeting HOXD9 therapeutically also warrants further validation. Overall, this work provides a foundation for future investigations to build upon, with the shared goal of improving prognosis for lethal ATC.

## Conclusions

The key insight from this research is that HOXD9 is a cancer-promoting factor that is significantly expressed in ATC and high levels of HOXD9 expression are associated with a dismal prognosis for survival of ATC patients. Mechanistically, HOXD9 binds to the miR-451a promoter and activates miR-451a transcription. HOXD9 induces miR-451a to directly target PSMB8, causing a switch from an apoptotic role to one that promotes migration, invasion, proliferation, and EMT characteristics in ATC. In vitro and in vivo results indicated that HOXD9/miR-451a/PSMB8 pathway plays a vital role in ATC regulation through the activation of the PI3K/AKT pathway. Our study indicates that HOXD9 interference could affect the progression and metastasis of ATC, and thus serve as a potential target for the diagnosis and management of this disease.

### Supplementary Information

**Additional file 1: Figure S1. **HOXD9 is abnormally upregulated in various cancers. **A** Analysis of the HOXD9 expression between tumor and normal tissues from the TCGA database. **B** By accessing to the TCGA and Genotype-Tissue Expression (GTEX), HOXD9 expression was found upregulated in THCA tissues. **C**, **D** Expression of HOXD9 in various subtypes of THCA, including 11 cases of ATC, 49 cases of PTC, and 45 cases of normal thyroid from the GEO database (GSE33630). **E** The survival plots of HOXD9 in TGCA-THCA. **Figure S2.** qRT-PCR and Western blot analysis were employed to assess the efficacy of interfering with and over-expressing HOXD9 in 8505c cells. **Figure S3. **HOXD9 promotes proliferation and metastasis by negatively regulating miR-451a *in vitro*.** A**, **B** Cell growth was determined by the CCK-8 assay. **C**, **D** Cell migration and invasion were assessed by Transwell assays. **E**, **F** The tumor cell-induced colony formation was also analyzed, and the colony formation rate was calculated. **G**, **H** Apoptosis of 8505c cells analyzed by flow cytometry. **I**, **J** The expression of E-cadherin and N-cadherin was detected through conducting immunofluorescence analysis. The data represent mean values ± SD, n = 3. **p < 0.01. **Figure S4. **PSMB8 is a direct target of miR-451a. **A**, **B** Correlation between PSMB8 with HOXD9 and miR-451a in THCA was analyzed on the ENCORI pan-cancer analysis platform using the expression data from the TCGA. **C** PSMB8 mRNA expression in normal samples and THCA tissues from the TCGA. **D** Association of PSMB8 mRNA expression with prognosis within the TCGA-THCA study. **E-G** Interference with PSMB8 expression in 8505c, qPCR (**A**) and WB (**B**, **C**) to detect interference efficiency. **Table S1. **Sequences for siRNA interference assay.

## Data Availability

The data supporting the conclusions of this article are included within the article.

## Abbreviations

ATC: Anaplastic thyroid carcinoma
HOXD9: Homeobox D9
miRNA: MicroRNA
PSMB8: Proteasome 20S subunit beta 8
HOX: Homeobox
HCC: Hepatocellular carcinoma
EMT: Epithelial–mesenchymal transition
GAPDH: Glyceraldehyde 3-phosphate dehydrogenase
CHIP: Chromatin immunoprecipitation
TCGA: The Cancer Genome Atlas
THCA: Thyroid carcinoma
PTC: Papillary thyroid carcinoma
GEO: Gene Expression Omnibus
GSEA: Gene set enrichment analysis
qRT-PCR: Real-time quantitative PCR
IVIS: In vivo imaging system
TUNEL: Transferase dUTP nick end labeling
IHC: Immunohistochemistry
OS: Overall survival
HRP: Horseradish peroxidase
