# Supplementary material for: HOXD9/miR-451a/PSMB8 axis is implicated in the regulation of cell proliferation and metastasis via PI3K/AKT signaling pathway in human anaplastic thyroid carcinoma
Source: J Transl Med. 2023 Nov 16;21:817. doi: 10.1186/s12967-023-04538-0 (PMC10652604; doi:10.1186/s12967-023-04538-0)
Supplement: Supplementary file 1 — Additional file 1: Figure S1. HOXD9 is abnormally upregulated in various cancers. A Analysis of the HOXD9 expression between tumor and normal tissues from the TCGA database. B By accessing to the TCGA and Genotype-Tissue Expression (GTEX), HOXD9 expression was found upregulated in THCA tissues. C, D Expression of HOXD9 in various subtypes of THCA, including 11 cases of ATC, 49 cases of PTC, and 45 cases of normal thyroid from the GEO database (GSE33630). E The survival plots of HOXD9 in TGCA-THCA. Figure S2. qRT-PCR and Western blot analysis were employed to assess the efficacy of interfering with and over-expressing HOXD9 in 8505c cells. Figure S3. HOXD9 promotes proliferation and metastasis by negatively regulating miR-451a in vitro. A, B Cell growth was determined by the CCK-8 assay. C, D Cell migration and invasion were assessed by Transwell assays. E, F The tumor cell-induced colony formation was also analyzed, and the colony formation rate was calculated. G, H Apoptosis of 8505c cells analyzed by flow cytometry. I, J The expression of E-cadherin and N-cadherin was detected through conducting immunofluorescence analysis. The data represent mean values ± SD, n = 3. **p < 0.01. Figure S4. PSMB8 is a direct target of miR-451a. A, B Correlation between PSMB8 with HOXD9 and miR-451a in THCA was analyzed on the ENCORI pan-cancer analysis platform using the expression data from the TCGA. C PSMB8 mRNA expression in normal samples and THCA tissues from the TCGA. D Association of PSMB8 mRNA expression with prognosis within the TCGA-THCA study. E-G Interference with PSMB8 expression in 8505c, qPCR (A) and WB (B, C) to detect interference efficiency. Table S1. Sequences for siRNA interference assay. [file 12967_2023_4538_MOESM1_ESM.docx]

# HOXD9/miR-451a/PSMB8 axis is implicated in the regulation of cell proliferation and metastasis via PI3K/AKT signaling pathway in human anaplastic thyroid carcinoma

Yong Zhong^1, *^, Fan Yu^2, *^, Ling Yang^1, *^, Yu Wang^3^, Lin Liu^1^, Chengyou Jia^1^, Haidong Cai^1^, Jianshe Yang^1^, Shiyang Sheng^1^, Zhongwei Lv^1, #^, Bo Wu^2, #^, Xiaoping Zhang^1, #^

^1^ Department of Nuclear Medicine, Shanghai Tenth People’s Hospital, Tongji University, Shanghai, China.

^2^ Center of Thyroid, Department of General Surgery, Shanghai Jiao Tong University Affiliated Sixth People’s Hospital, Shanghai China.

^3^ Department of Head and Neck Surgery, Fudan University Shanghai Cancer Center, Shanghai, China.

**^*^These authors contributed equally to this article.**

**^#^Corresponding authors:**

**Bo Wu**: Center of Thyroid, Department of General Surgery, Shanghai Jiao Tong University Affiliated Sixth People’s Hospital, Shanghai 200233, China. Email: wubo7421@sohu.com

**Zhongwei Lv**: Department of Nuclear Medicine, Shanghai Tenth People's Hospital, Tongji University and Shanghai Center of Thyroid Diseases, No. 301 Middle Yanchang Road, Shanghai 200072, China. Email: shtjnmd@163.com

**Xiaoping Zhang:** Department of Nuclear Medicine, Shanghai Tenth People’s Hospital, Tongji University, Shanghai, 200072, China. Email: zxpkxy@tongji.edu.cn

**This PDF file includes: 1. Supplementary Figures. S1 to S4; 2. Supplementary Tables. S1 and S2.**

**Supplementary Figures and Figure legends**


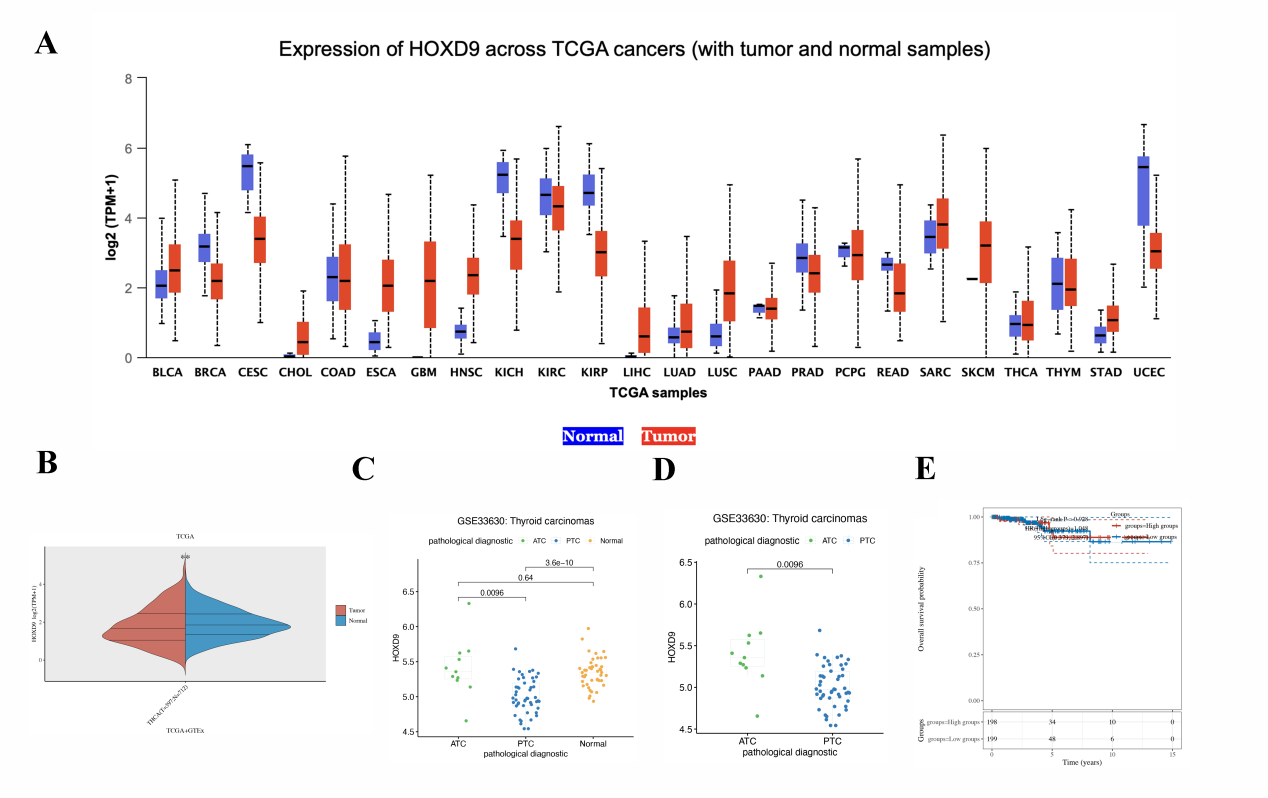


**Figure S1. HOXD9 is abnormally upregulated in various cancers.** (**A**) Analysis of the HOXD9 expression between tumor and normal tissues from the TCGA database. (**B)** By accessing to the TCGA and Genotype-Tissue Expression (GTEX), HOXD9 expression was found upregulated in THCA tissues. **(C, D**) Expression of HOXD9 in various subtypes of THCA, including 11 cases of ATC, 49 cases of PTC, and 45 cases of normal thyroid from the GEO database (GSE33630). (**E**) The survival plots of HOXD9 in TGCA-THCA.


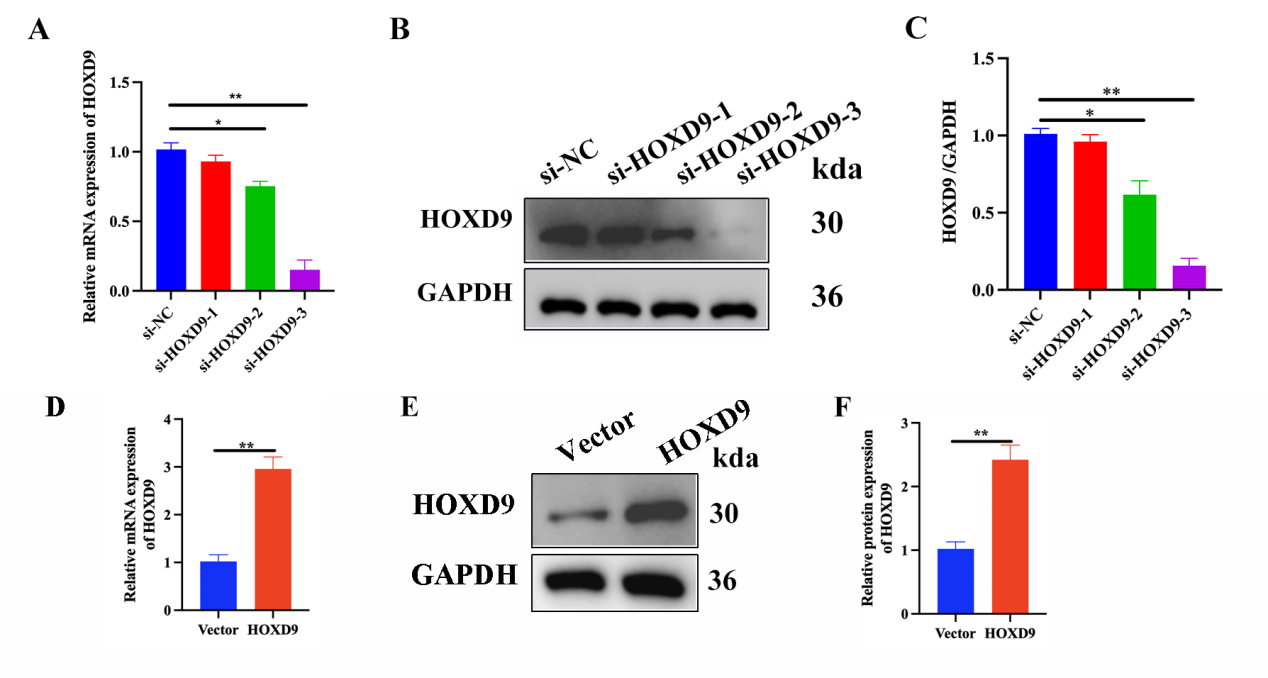


**Figure S2. qRT-PCR and Western blot analysis were employed to assess the efficacy of interfering with and over-expressing HOXD9 in 8505c cells.**

**
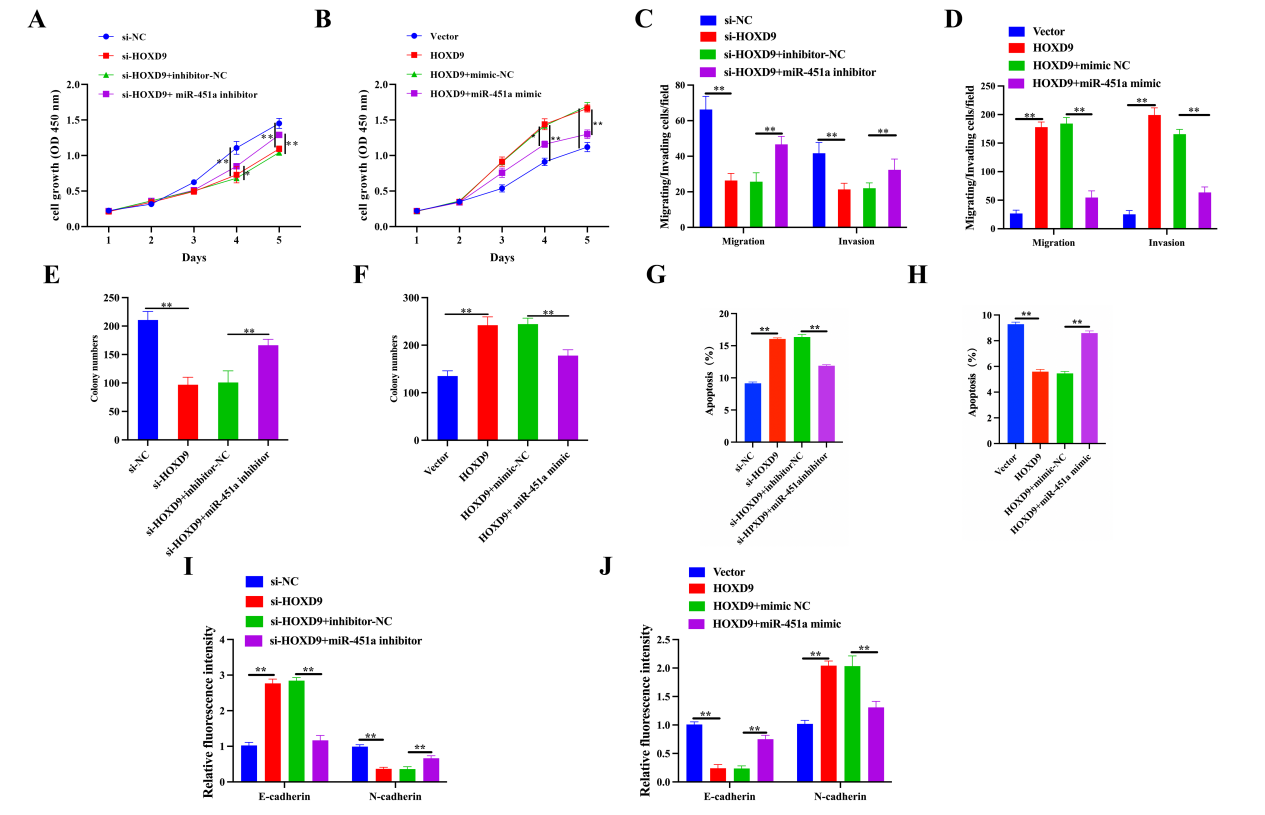
**

**Figure S3. HOXD9 promotes proliferation and metastasis by negatively regulating miR-451a *in vitro*.** (**A, B**) Cell growth was determined by the CCK-8 assay. (**C, D**) Cell migration and invasion were assessed by Transwell assays. (**E, F**) The tumor cell-induced colony formation was also analyzed, and the colony formation rate was calculated. (**G, H**) Apoptosis of 8505c cells analyzed by flow cytometry. (**I, J**) The expression of E-cadherin and N-cadherin was detected through conducting immunofluorescence analysis. The data represent mean values ± SD, n = 3. **p < 0.01.


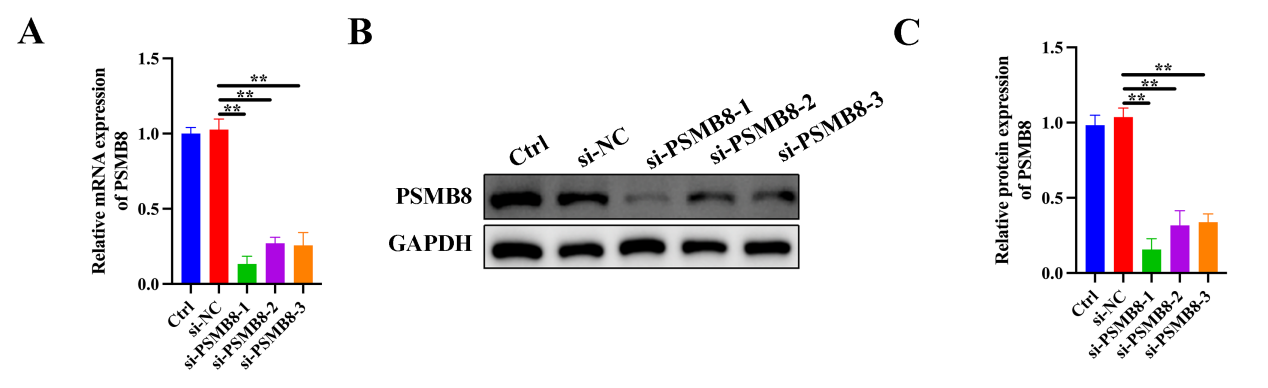


**
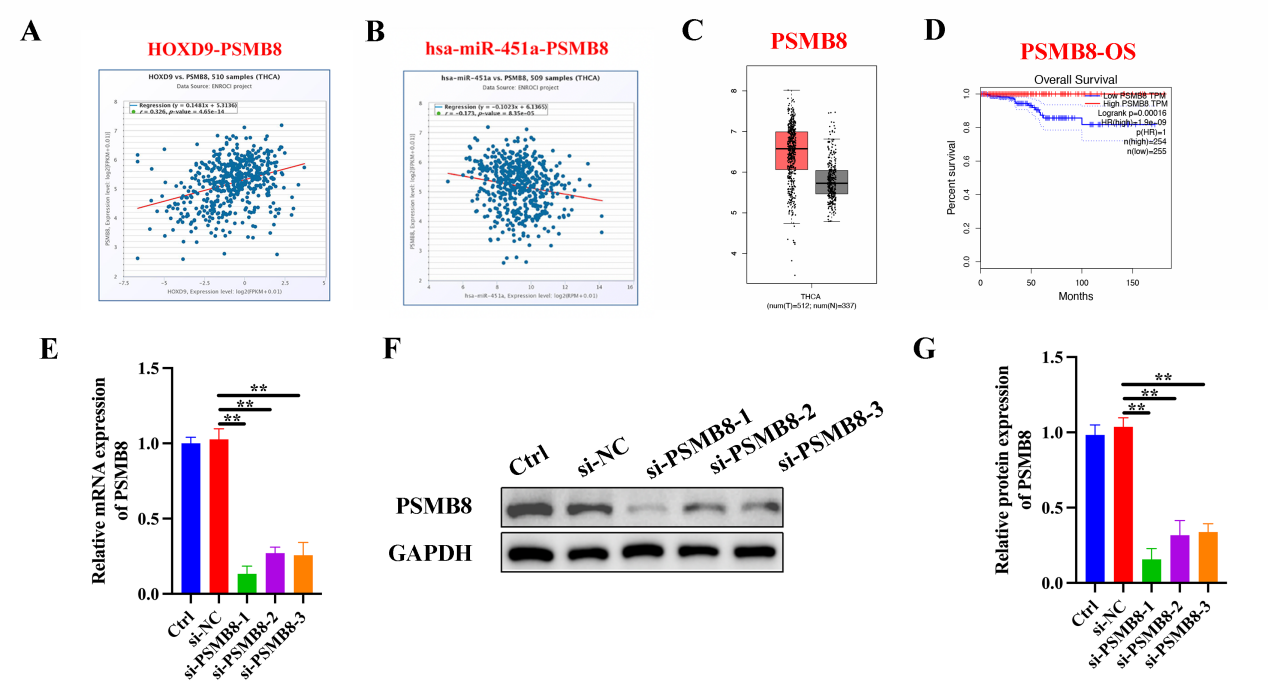
**

**Figure S4. PSMB8 is a direct target of miR-451a.** (**A, B**) Correlation between PSMB8 with HOXD9 and miR-451a in THCA was analyzed on the ENCORI pan-cancer analysis platform using the expression data from the TCGA. (**C**) PSMB8 mRNA expression in normal samples and THCA tissues from the TCGA. (**D**) Association of PSMB8 mRNA expression with prognosis within the TCGA-THCA study. (**E-G**) Interference with PSMB8 expression in 8505c, qPCR (A) and WB (B, C) to detect interference efficiency.

**Supplementary Tables**

**Table S1. Sequences for siRNA interference assay.**

| **Genes** | **Serial number** | **Sense (5’-3’)** | **Antisense (5’-3’)** |
| --- | --- | --- | --- |
| HOXD9 | 1# | GGACCAGUUGUAAAUGUUACU | UAACAUUUACAACUGGUCCUC |
|  | 2# | GGUGCUUGAUUUCCAGAAACU | UUUCUGGAAAUCAAGCACCAA |
|  | 3# | GGUUCCUUAAGAAAUGCUAUA | UAGCAUUUCUUAAGGAACCAA |
| E-cadherin | 1# | UGUAUUCAGCGUGACUUUGGU | CAAAGUCACGCUGAAUACAGU |
|  | 2# | UAGGAAAUGGGCCUUUUUCAU | GAAAAAGGCCCAUUUCCUAAA |
|  | 3# | UUCUCUUUCAAUAAUAAAGAC | CUUUAUUAUUGAAAGAGAAAC |
| PSMB8 | 1# | UGGAUUUGUACCAUUCUUCUG | GAAGAAUGGUACAAAUCCAAG |
|  | 2# | ACUCAUUGGUUCCUUUAAGGG | CUUAAAGGAACCAAUGAGUCC |
|  | 3# | UCAUAUUCUGAAUCUCAUCCU | GAUGAGAUUCAGAAUAUGAAG |
| Si-NC | - | UUCUCCGAACGUGUCACGU | ACGUGACACGUUCGGAGAA |
